# A Selective Copper Based Oxygen Reduction Catalyst for the Electrochemical Synthesis of H_2_O_2_ at Neutral pH

**DOI:** 10.1002/celc.202101692

**Published:** 2022-02-02

**Authors:** Bas van Dijk, Rick Kinders, Thimo H. Ferber, Jan P. Hofmann, Dennis G. H. Hetterscheid

**Affiliations:** ^1^ Leiden Institute of Chemistry Leiden University P. O. Box 9502 2300 RA Leiden, The Netherlands; ^2^ Surface Science Laboratory Department of Materials and Earth Sciences Technical University of Darmstadt Otto-Berndt-Strasse 3 64287 Darmstadt Germany

**Keywords:** copper complexes, electrocatalysis, homogeneous catalysis, hydrogen peroxide, oxygen reduction

## Abstract

H_2_O_2_ is a bulk chemical used as “green” alternative in a variety of applications, but has an energy and waste intensive production method. The electrochemical O_2_ reduction to H_2_O_2_ is viable alternative with examples of the direct production of up to 20% H_2_O_2_ solutions. In that respect, we found that the dinuclear complex Cu_2_(btmpa) (6,6’‐bis[[bis(2‐pyridylmethyl)amino]methyl]‐2,2’‐bipyridine) reduces O_2_ to H_2_O_2_ with a selectivity up to 90 % according to single linear sweep rotating ring disk electrode measurements. Microbalance experiments showed that complex reduction leads to surface adsorption thereby increasing the catalytic current. More importantly, we kept a high Faradaic efficiency for H_2_O_2_ between 60 and 70 % over the course of 2 h of amperometry by introducing high potential intervals to strip deposited copper (^dep^Cu). This is the first example of extensive studies into the long term electrochemical O_2_ to H_2_O_2_ reduction by a molecular complex which allowed to retain the high intrinsic selectivity of Cu_2_(btmpa) towards H_2_O_2_ production leading to relevant levels of H_2_O_2_.

## Graphical abstract

**Optimised catalytic activity**: Electrochemical H_2_O_2_ production is a promising sustainable alternative to the anthraquinone method. In that perspective, we have studied the dinuclear copper complex Cu_2_(btmpa) that has a high intrinsic H_2_O_2_ selectivity. By extensive, long term studies, we found that complex accumulation on the electrode increases the catalytic activity and that high potential intervals strip away deposited copper. Through this procedure we were able to reach a 60 to 70 % Faradaic efficiency during long term amperometry experiments.

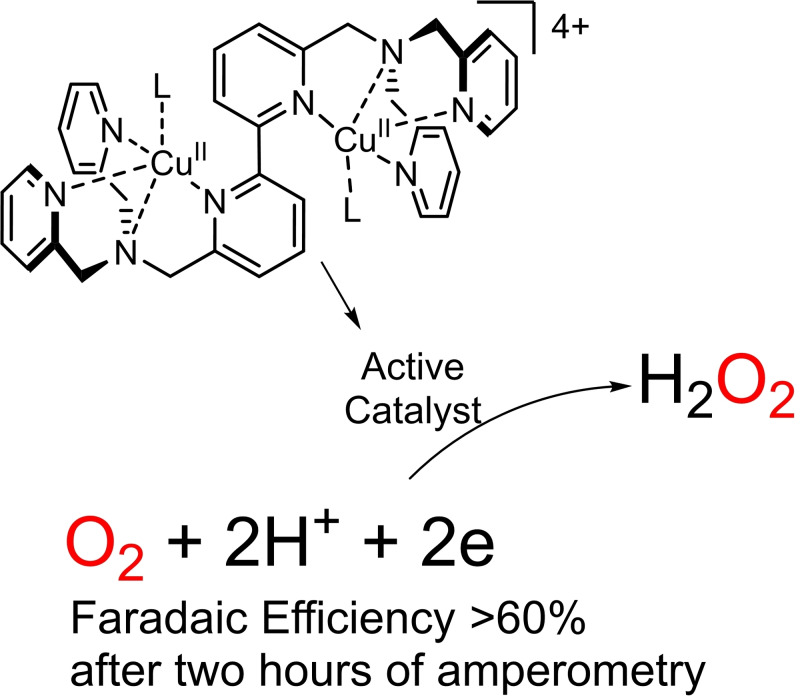

## 1 Introduction

H_2_O_2_ is a bulk chemical that is produced on a 4.5 million ton scale^[1]^ and used in many applications^[2]^ such as bleaching (largest single use),^[3]^ waste water treatment,^[4]^ disinfecting, and industrial organic synthesis.^[5]^ It is considered as an environmentally friendly chemical oxidant because the decomposition products are water and/or O_2_. However, its current production method is far from environmentally friendly. Over 90 % of the worldwide H_2_O_2_ production proceeds via the anthraquinone process.[^2^, ^6^] Here, anthraquinones are used as redox mediators that first undergo reduction with H_2_, followed by a separate re‐oxidation in the presence of air (O_2_) which produces H_2_O_2_ selectively. Liquid‐liquid extractions are required to extract H_2_O_2_ given that these reactions take place in organic solvent. Consequently, the obtained H_2_O_2_ is contaminated with organic impurities. As a result, most of the cost and energy of producing H_2_O_2_ result from the purification of this extract.

The electrochemical reduction of O_2_ to H_2_O_2_ is a viable alternative and was first reported in 1939 by Berl.^[7]^ In fact, it has been industrialized in the Huron‐Dow process which is mostly used for on‐site production of alkaline peroxide mixtures for the paper bleaching industry. Nevertheless, this only covers a negligible fraction of the total H_2_O_2_ production.[^2^, ^6^] To overcome the problem of separating the H_2_O_2_ from the aqueous electrolyte, solid electrolyte cells in combination with flow cell chemistry have recently been proposed as a feasible option.^[8]^ The cathode, where O_2_ reduction takes place, can be made of several materials. Noble metals usually catalyze the full 4 electron reduction to H_2_O or they interact weakly with O_2_ resulting in low rates and a high overpotential. Alloys combine these characteristics and result in better catalysts.^[9]^ Another interesting approach is the use of carbon based electrodes because these have an intrinsic selectivity towards the formation of H_2_O_2_ when performing O_2_ reduction.^[10]^ Nevertheless, their reactivity is quite poor, and application of such materials therefore requires large overpotentials.^[9a]^ Improvements can be made by increasing the defect^[11]^ and/or oxygen content,^[12]^ doping with heteroatoms,^[13]^ or doping with metals as single‐site catalysts. The latter approach is challenging since metal–support interactions for carbon are relatively weak.^[9a]^ In those cases, molecular complexes can improve adsorption through the ligand‐carbon interactions. Most molecular catalysts, that have been reported to perform the reduction of O_2_ to H_2_O_2_, have only been studied in non‐aqueous solvents.^[14]^ Mechanisms and selectivity depend significantly on the acid type and acid strength and cannot be directly translated to aqueous solutions. Until now, high selectivity for electrocatalytic H_2_O_2_ production in aqueous solutions is observed only for a few manganese,^[15]^ iron,^[16]^ copper,[^15b^, ^17^] and cobalt complexes.[^15b^, ^16d^, ^18^] The initial high selectivity for H_2_O_2_ is generally restricted to a small potential window and only observed for a few minutes. Longer measurements are rarely performed and if so, they typically result in an overall 4 electron selectivity either due to over‐reduction of H_2_O_2_ or due to the disproportionation of H_2_O_2_, also catalyzed by the same molecular catalysts.^[16b]^ Thus far, there is only the exception of a cobalt tetrakis(*N*‐methyl‐4‐pyridyl)porphyrin complex that was reported with high selectivity (>90 %) for H_2_O_2_ after 2 h of electrolysis, but no further details were provided to support this claim.^[18a]^

**Scheme 1:**
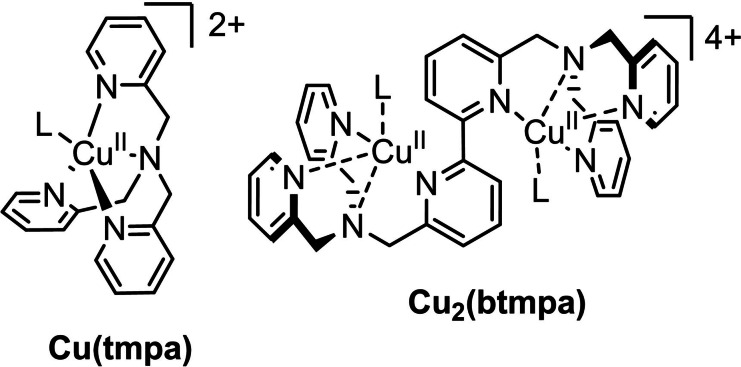
Structures of Cu(tmpa) and Cu_2_(btmpa). L=H_2_O in an aqueous solution, and probably rapidly exchanging with phosphate in a phosphate buffer.

Our group reported [Cu(tmpa)(L)]^2+^ (Cu(tmpa) (Scheme 1), tmpa=tris(2‐pyridylmethyl)amine, L=solvent) for the electrochemical 4 electron reduction of O_2_ to H_2_O that proceeds in a stepwise mechanism with H_2_O_2_ as detectable intermediate.^[17]^ At pH 7, two separate catalytic cycles for O_2_ to H_2_O_2_ and H_2_O_2_ to H_2_O reduction take place with onsets of 0.50 and 0.45 V *versus* the reversible hydrogen electrode (RHE), respectively. Cu(tmpa) is an intrinsic very fast catalyst for the O_2_ to H_2_O_2_ reduction, but over‐reduction of H_2_O_2_ to H_2_O is fast as well. For that reason, we set out to investigate the dicopper complex [Cu_2_(btmpa)(L)_4_]^4+^ (Cu_2_(btmpa), btmpa=6,6’‐bis[[bis(2‐pyridylmethyl)amino]methyl]‐2,2’‐bipyridine) which is consisting of two Cu(tmpa) moieties fused via a covalent bond between one of the three pyridines on each moiety resulting in a bipyridine backbone (Scheme 1).^[19]^ An earlier report suggested that the Cu^I^ complex [Cu_2_(btmpa)(MeCN)_2_(ClO_4_)_2_]^2+^ has a Cu^I^ geometry that was different from Cu^I^(tmpa), consequently a positively shifted redox potential of the Cu^II/I^ redox couple compared to Cu(tmpa). As a result isolated and well‐characterized [Cu^I^_2_(btmpa)](ClO_4_)_2_ shows a diminished reactivity towards O_2_ with respect to Cu(tmpa) in acetonitrile.^[19c]^ We found that these changes in geometric properties in particular affected the electrochemical H_2_O_2_ reduction resulting in a high selectivity up to 100 % for H_2_O_2_. In addition, we performed the first systematic study of long bulk electrosynthesis of H_2_O_2_ by a molecular catalyst. By performing long term amperometry measurements, we were able to identify factors that limit the Faradaic efficiency; were able to improve the overall electrosynthesis process, and thereby achieved a record breaking Faradaic efficiency of 65 % for H_2_O_2_ over the course of 2 h.

## 2 Results and Discussion

### 2.1 The (electronic) structure of Cu_2_(btmpa)

The dinuclear complex Cu_2_(btmpa) was synthesized from the btmpa ligand and Cu(OTf)_2_, while the ligand was synthesized according to an earlier reported synthesis that was slightly adjusted to increase the purity and yield (see supporting info).^[19b]^ EPR and SQUID measurements did not show a large coupling interaction between the two Cu^2+^ centers of the complex (Figures S1 and S2). We found the copper centers of Cu_2_(btmpa) are reduced simultaneously in an 0.1 m phosphate buffer solution of pH 7 (Figure S3), similar to earlier studies in organic solvents.^[19c]^ In addition, the Cu^II/I^ redox potential shifted 0.3 V positively with respect to the mononuclear Cu(tmpa) towards 0.51 V *versus* the Reversible Hydrogen Electrode (RHE). A previously published crystal structure of a [(btmpa)Cu_2_(CH_3_CN)_2_(ClO_4_)_2_]^2+^ complex showed that the Cu−N bond of the bipyridine moiety has longer distances (2.4 Å) than the other pyridines (2.0 Å).^[19c]^ As a result, the Cu^II^ site is likely less electron dense than Cu(tmpa) which explains the positive shift of the Cu^II/I^ redox couple. The Cu^II/I^ redox couple of Cu_2_(bmpta) has a relatively large peak separation, which increases with increasing scan rate (Figure S3B). In line with Marcus theory, in which a higher reorganization energy is linked to slower electron transfer,^[20]^ this points to a relative slow electron transfer process. In contrast to Cu_2_(btmpa), the reduction of the mononuclear Cu(tmpa) complex is a very fast process^[17]^ due to the easy transition of a trigonal bipyramidal geometry of the Cu^II^ complex to the preferred tetragonal geometry for the Cu^I^ state by the elongation of Cu−N distance of the tertiary amine from 2.10 to 2.43 Å.^[21]^ In contrast, the Cu^II^ geometry of Cu_2_(btmpa) leans towards a more pseudo‐octahedral geometry,^[19c]^ and it seems unlikely that Cu_2_(btmpa) can easily obtain the preferred tetragonal geometry for the Cu^I^ state during redox state changes.

### 2.2 Electrocatalysis in presence of Cu2(btmpa)

**Figure 1:**
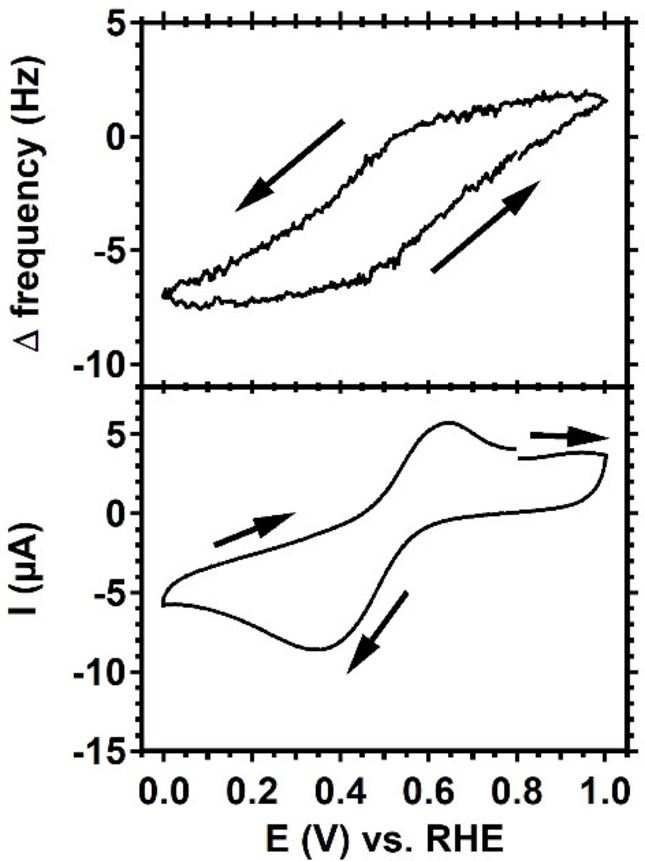
Electrochemical quartz crystal microbalance measurement with a gold work electrode of 0.15 mm Cu_2_(btmpa) in 0.1 m phosphate buffer of pH 7. The bottom panel shows the second scan of a CV at 50 mV/s scan rate under argon atmosphere. The top panel shows the relative frequency of the quartz crystal and its response with respect to the applied potential.

During reduction from the +II to the +I oxidation state Cu_2_(btmpa) has a tendency to adsorb on the electrode. This behavior was studied in detail with electrochemical quartz crystal microbalance (EQCM) studies (Figure 1 and Figure S4), which showed that the reduction of Cu_2_(btmpa) from a total 4+charge to 2+charge does trigger adsorption on the electrode, whereas electrochemical oxidation triggers desorption. Although the EQCM data show that the potential‐dependent adsorption is reversible on gold electrodes, the carbon‐based GC electrode might have a stronger affinity with Cu_2_(btmpa).

**Figure 2:**
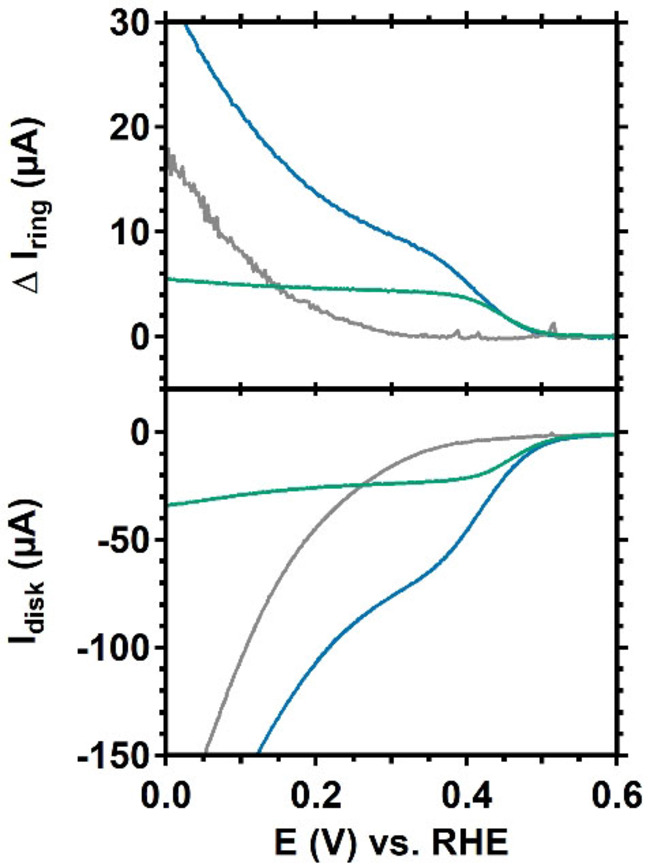
Linear sweep voltammograms with a rotating ring disk electrode setup of 0.15 mm Cu_2_(btmpa) under argon (green) and O_2_ atmosphere (blue). The cyclic voltammogram of the GC disk (bottom panel) and the current response of the Pt ring (top panel) are shown. The grey line represents the GC disk in catalyst‐free, O_2_ purged electrolyte. The voltammograms were recorded at 50 mV/s in a 0.1 m phosphate buffer of pH 7. A rotation rate of 1600 rpm and a Pt ring potential of 1.2 V were applied.

Studies with a rotating ring disk electrode (RRDE) setup of the O_2_ reduction reaction (ORR) showed that Cu_2_(btmpa) reduces O_2_ to H_2_O_2_ with an onset of 0.50 V *versus* RHE (Figure 2). At potentials lower than 0.35 V, the GC electrode itself reduces O_2_ to H_2_O_2_ as well (Figure S5) and increases the reductive current when performing cyclic voltammetry (CV).Compared to Cu(tmpa) the O_2_ reduction and in particular the H_2_O_2_ reduction reactions mediated by Cu_2_(btmpa) are slow (see supporting info). This is in line with the electron transfer rates being significantly slower as well in case of Cu_2_(btmpa). In addition the binding affinity of dioxygen are also lower, which is most likely due to the copper +I oxidation state being relatively stable, illustrated by the higher E_1/2_ value of Cu_2_(bmpta) compared to Cu(tmpa). The slow H_2_O_2_ reduction in presence of Cu_2_(btmpa) results in a relatively high selectivity towards hydrogen peroxide. Whereas the rate determining step in case of the oxygen reduction reaction mediated by Cu(tmpa) is binding of dioxygen,^[17]^ the rate determining step of hydrogen peroxide reduction most likely involves cleavage of the O−O bond via a Fenton like mechanism.^[22]^ It is not unlikely that the reaction rate of the more complex reaction step is slowed down the most by more problematic electron transfer. Although we cannot rule out that activation of the btmpa‐ligand initiated by reactive oxygen species (ROS) produced by either the copper species and/or the carbon electrode may play a role as well in these observations.

Since Cu_2_(btmpa) adsorbs at the electrode when negative potentials are applied, this allows one to significantly increase the number of active copper sites at the electrode interface over time and thereby strongly increase the peroxide productivity during amperometry measurements. Chronoamperometry in presence of Cu_2_(btmpa) was performed using a rotating ring disk setup (Figure S6B). A $N_{CE}$ (collection efficiency of the ring electrode) of 17.5 % was determined and used to calculate the % H_2_O_2_ for this measurement (see Figures S7 and S8 for the method). The selectivity for H_2_O_2_ production by Cu_2_(btmpa) initially starts at 90 %. Over the course of 15 minutes, the selectivity lowers to 70 %. A selectivity below 100 % suggests that over‐reduction of H_2_O_2_ takes place. For that purpose, H_2_O_2_ reduction by Cu_2_(btmpa) under argon atmosphere was studied with non‐rotating and rotating electrodes (Figure S9). H_2_O_2_ is reduced by Cu_2_(btmpa) indeed and the reducing current increases with the H_2_O_2_ concentration. However, the H_2_O_2_ reduction by Cu_2_(btmpa) is very sluggish which explains the high selectivity for H_2_O_2_ when performing O_2_ reduction. When chronoamperometry measurements were performed for a longer period, a significant drop in selectivity was observed. This is most likely linked to formation of deposited copper (^dep^Cu) at the cathode because a brown‐colored, metallic deposit could be observed on the surface of the electrode (Figure S10). Formation of this copper deposit inherently changes the selectivity from H_2_O_2_ to H_2_O over the course of time. To counter formation of ^dep^Cu we applied stripping intervals, wherein the potential at the working electrode is periodically increased to 0.8 V vs RHE, which is sufficient to strip ^dep^Cu from the electrode, yet insufficient to oxidize H_2_O_2_ itself (Figure S9).

### 2.3 Employing stripping intervals to produce hydrogen peroxide over several hours

**Figure 3:**
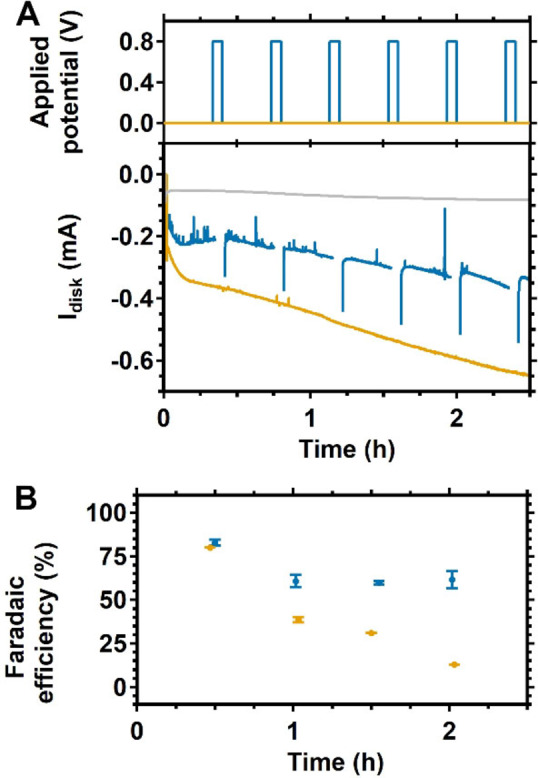
Rotating disk chronoamperometry of a GC disk at 0.0 V in a O_2_ saturated Cu_2_(btmpa) solution in a continuous measurement (orange) or a 20 minute interval measurement (blue). For the latter, a 0.8 V potential was applied for 4 minutes to re‐oxidize adsorbed Cu^0^ deposition every 20 minutes according to the sequence shown in panel (A). The Faradaic efficiency for H_2_O_2_ is given in (B). The black dots represent the Faradaic efficiency of the time window since the last H_2_O_2_ measurement. The disk was rotated at 1600 rpm in a 0.1 m phosphate buffer of pH 7 with 0.15 mm catalyst.

O_2_ reduction with Cu_2_(btmpa) was monitored over a 2 hour period in O_2_ saturated phosphate buffer. To do so, a rotating disk setup was used for constant diffusion of O_2_ saturated electrolyte at 1600 rpm rotation rate. We chose 0.0 V as the most ideal potential because a background hydrogen evolution reaction is not to be expected here, and background O_2_ reduction reactions on GC are still minimal at this potential, while significant currents were generated at this potential in presence of Cu_2_(btmpa) in amperometry experiments. Three different types of measurements were performed (Figure 3). First, a GC electrode in absence of Cu_2_(btmpa) was tested as blank measurement (grey line). Second, a GC electrode in 0.15 mM catalyst solution was tested while continuously applying 0.0 V (orange). Last, a GC electrode in catalyst solution was tested with intervals (blue): after 20 minutes of 0.0 V, the potential at the disk was briefly set at 0.8 V for 4 minutes (see the scheme in the top panel of Figure 3. The results of the continuous measurements with and without catalyst show that the current is significantly higher in the presence of Cu_2_(btmpa) (−0.25 mA *versus* −0.05 mA in the first minutes) and increases gradually over the course of 2 h. In the first half hour, there is a large increase in current from −0.25 to −0.37 mA. This feature of quick increase within the first 30 minutes of the measurement is observed in all cases when catalyst is present but not in absence of the catalyst. At 0.0 V, O_2_ reduction by Cu_2_(btmpa) is still kinetically limited in a cyclic voltammetry experiment (Figure S5). As a result, the large increase in reductive current can be explained by an increase in active sites due to accumulation of the catalyst on the electrode. The EQCM measurements points towards this behavior as well (Figure 1). While the electrochemical production of hydrogen peroxide appears to be catalyzed by catalytic material adsorbed on the electrode interface, it seems likely that it is the adsorbed Cu_2_(btmpa) complex, or a reaction product thereof that still retains some form of an organic ligand, that is responsible for the two‐electron reduction of dioxygen. Heterogeneous copper species, either with Cu in the +0 oxidation state, or in a partly oxidized form are not expected to produce hydrogen peroxide in significant concentrations.^[22]^

Interestingly, the magnitude of the current at 0.0 V after a 4‐minute 0.8 V interval is equal to the magnitude of the final part of the preceding 20 minutes amperogram. This indicates that the adsorbed Cu_2_(btmpa) largely retains on the GC electrode even when a potential of 0.8 V is briefly applied. Only thoroughly rinsing the electrode could remove most of the adsorbed catalyst and lower the O_2_ reduction current to the same level of a bare GC electrode in a catalyst‐free electrolyte (see Figure S11). After 1.5 hours, the reducing current of the continuous measurement became close to the estimated diffusion limited current (−0.49 mA) that one would expect for reduction of O_2_ to H_2_O_2_ under these conditions. The estimation is based on the diffusion limited current that a Pt disk of the same size (0.196 cm^2^) reaches under the same conditions for the 4‐electron reduction of O_2_ to H_2_O, for which −0.98 mA was obtained.^[17]^ However, the current continues to rise even further in longer measurements (Figure S13), pointing to a significant overreduction of H_2_O_2_.

When O_2_ reduction at 0.0 V was alternated with short periods of ^dep^Cu stripping at 0.8 V (blue line of Figure 3) the magnitude of the reducing current at 0.0 V is lower as compared to the continuous measurement suggesting that less over‐reduction of H_2_O_2_ takes place. Visibly, this interval procedure prevents the over‐reduction by the Cu deposition to a certain extent with respect to a continuous measurement.

The Faradaic efficiency for H_2_O_2_ at 0.0 V was monitored to study the effect of over‐reduction of H_2_O_2_ by either Cu_2_(btmpa) or ^dep^Cu. Two different methods were considered. The first method used the same RRDE set‐up as used for Figure S6 that utilizes the Pt ring as electrochemical H_2_O_2_ sensor. Here, we found that the ring is not suited as quantitative peroxide sensor during long‐term electrolysis (see Figure S12). The formation of high amounts of H_2_O_2_ results in oxidation of Pt to produce PtO*x* resulting in deactivation of the activity of the Pt ring. (Figures S7 and S8).^[23]^ However, the data did suggest that there was a slow build‐up of H_2_O_2_ within the reaction mixture. Therefore, we applied a second method: bulk electrolysis with an RDE setup for which the bulk concentration of H_2_O_2_ was periodically determined with an enzyme based photometric analysis using a reflectometer. The Faradaic efficiency was determined for measurements with 4 minute intervals and no intervals of 0.8 V. The results of the 4‐minute interval and continuous measurement are shown in Figure 3B. Within the first 30 minutes, a Faradaic efficiency of 83 % was obtained which is in good agreement with the selectivity that was found with the 15 minute RRDE measurement at 0.2 V (Figure S6). Likewise, in the continuous measurement without intervals, an efficiency of 80 % was found after the first 30 minutes (Figure 3). The continuous measurement showed a drastic drop in efficiency to 40 % 1 hour after the start and stagnated around 10 % after 2 h. The Faradaic efficiencies remained at 60 to 70 % during the interval experiments in the same time window clearly indicating that the interval procedure greatly enhances the Faradaic efficiency. Typically, peroxide concentrations between 0.15 mM (after 2 h) and 0.5 mM (after 8 hours) are obtained, which are of sufficient concentration of a substantial number of direct applications as anti‐bacteria and anti‐algae reagent. Measurements for longer than 2 h showed that the formation of ^dep^Cu starts to decrease the Faradaic efficiency also in case of experiments with intervals (Figure S13 and S14). Here, it appears that the rising H_2_O_2_ concentration leads to a faster formation of ^dep^Cu. Most likely, the ligand is (partially) oxidized that leads to degradation of the complex (see supporting information). XPS measurements of the electrode post catalysis confirm the presence of a copper species different from Cu_2_(btmpa) on the electrode (Figure S17). Experiments wherein the electrolyte solution was spiked deliberately with hydrogen peroxide showed significant levels of overreduction, while treatment of Cu_2_(btmpa) with H_2_O_2_ resulted to visual color changes that can be attributed to ligand oxidation.

### 2.4 Pinpointing the activity to an active species

Cu_2_(btmpa) was found to adsorb reversibly at the electrode, and as more material deposits the activity of the ORR increases. Simultaneously the Faradaic efficiency towards formation of H_2_O_2_ decreases from >80 to >60 % during this stage. After several hours the catalytic currents increase, mostly due to more efficient reduction reaction of hydrogen peroxide, resulting in a decrease in Faradaic efficiency. Selectivity of the deposited material can be restored by stripping ^dep^Cu from the electrode. These observations point to a gradual degradation process of adsorbed Cu_2_(btmpa) to ^dep^Cu. Reactive oxidation species generated from O_2_ and in particular H_2_O_2_ are likely to play a role herein. We recently have shown that the reduction of peroxide mediated by Cu(tmpa) most likely proceeds via a Fenton like mechanism wherein ROS are involved.^[22a]^ It is unclear which species along the path from Cu_2_(btmpa) to ^dep^Cu is responsible for the high ORR rates leading to selective formation of H_2_O_2_. However, it is clear that this is not the final species in the sequence. Control experiments with Cu^2+^ salts do not lead to significant amounts of peroxide being formed,^[23]^ suggesting that the active species at least contains some form of an organic ligand. The degradation of the active species, however, can be largely prevented by interception of H_2_O_2_. Studies with an RRDE setup (Figure S12) wherein the H_2_O_2_ is continuously reduced to O_2_ by the platinum ring displayed a high selectivity and stable catalytic rates throughout an entire 8 hour measurement, which would make these systems very suitable for flow cell chemistry.

## 3 Conclusion

We have shown that significant amounts of hydrogen peroxide can be produced during long term amperometry experiments employing a copper catalyst. Due to accumulation of the reduced catalysts on the electrode surface, sufficiently high currents could still be obtained which are very close to the mass transport limited currents that one in principle could reach for a two electron reduction reaction involving dioxygen with the RRDE setup of study. Periodic stripping of the cathode is important for long term selectivity, as it removes ^dep^Cu formation at the electrode interface. We anticipate that our results will allow for the next step, which is the incorporating the catalyst in electrochemical flow cell devices for the direct electrochemical production of H_2_O_2_ from O_2_.

## Conflict of interest

The authors declare no conflict of interest.

4

## Supporting information

As a service to our authors and readers, this journal provides supporting information supplied by the authors. Such materials are peer reviewed and may be re‐organized for online delivery, but are not copy‐edited or typeset. Technical support issues arising from supporting information (other than missing files) should be addressed to the authors.

Supporting Information

## Data Availability

The data that support the findings of this study are available in the supplementary material of this article.
