# Supplementary material for: A Selective Copper Based Oxygen Reduction Catalyst for the Electrochemical Synthesis of H2O2 at Neutral pH
Source: ChemElectroChem. 2022 Feb 2;9(3):e202101692. doi: 10.1002/celc.202101692 (PMC9305592; doi:10.1002/celc.202101692)
Supplement: Supplementary file 1 — Supporting Information [file CELC-9-0-s001.pdf]

ChemElectroChem

Supporting Information

# A Selective Copper Based Oxygen Reduction Catalyst for the Electrochemical Synthesis of $H_2O_2$ at Neutral pH

Bas van Dijk, Rick Kinders, Thimo H. Ferber, Jan P. Hofmann, and Dennis G. H. Hetterscheid*

# Experimental

## General

All chemicals were bought from commercial suppliers and used as received without further purification. The pH was measured with a Hannah Instruments HI 4222 pH meter that was calibrated with five IUPAC standard buffers. UV-vis measurements were performed on a Varian Cary 50 UV-vis spectrometer. $^1H$ NMR measurements were performed with a Bruker DPX-300 spectrometer using deuterated solvents obtained from Eurisotop. $CDCl_3$ was purified by filtration over basic alumina before use. All chemical shifts (δ) are reported with respect to the solvent peak.[1] Elemental analysis was performed by Mikroanalytisches Laboratorium Kolbe in Oberhausen, Germany. Mass spectra were recorded on a Thermo Fisher Scientific MSQ Plus ESI. EPR spectra were recorded on a Bruker EMXplus X-band spectrometer. Fitting of the EPR spectra was performed with W95EPR software developed by Prof. Dr. Frank Neese. Reflectance Raman spectra were recorded with a WITech alpha 300 R confocal Raman microscope equipped with a 532 nm laser (1 mW). A 100x objective with a numerical aperture of 0.9 was used (Zeiss). For preparation of the sample, an aqueous solution of 3 mM **$Cu_2$(btmpa)** and 22 mM $H_2O_2$ was dropcasted on a $Si/SiO_2$ wafer (Siegert wafer) with a native silicon oxide layer of 285 nm. SQUID measurements were performed on a Quantum Design MPMS-XL 7T SQUID magnetometer employing the settle approach with activated No-overshoot mode om the 300-2K temperature range with varying increment: 5K (300-150K), 2K (150-50K), 1K (50-25K), and 0.5K (25-2K) at constant 5 kOe magnetic field. An automatic diamagnetic correction was applied for a sample holder that was measured separately beforehand. Each data point was averaged over 4 consecutive scans.

## Synthesis

The dinucleating btmpa ligand was synthesized by the $S_N2$ reaction of 6,6'-(dichloromethyl)-2,2'-bipyridine (**4**) and commercially available 2,2'-dimethylpyridylamine (dmpa) following literature proceedings.[2] The earlier reported synthesis was slightly adjusted by using **4** which bears a chloromethyl instead of bromomethyl moiety (Scheme S1). We found that unreacted dmpa was hard to remove by common chromatography methods as both btmpa and dmpa display significant tailing. Therefore, an additional purification method was developed to improve the yield of pure btmpa (for details, see below). In short, dmpa is selectively converted to an amide by adding hexanoic anhydride to the crude mixture of dmpa and **4**. This amide can easily be removed by chromatography methods and btmpa was successfully purified with a yield of 21%. Next, the $Cu^{II}$ complex was synthesized by mixing a solution of $Cu^{II}(CF_3SO_3)_2$, and btmpa. After recrystallization by vapor diffusion of diethyl ether in a methanolic solution, blue colored crystals of the complex were obtained with 63% yield with $[Cu_2(btmpa)(CH_3OH)_2](CF_3SO_3)_4$ as molecular formula determined by elemental analysis. Neutral aqueous solutions containing **$Cu_2$(btmpa)** are blue colored arising from a broad $Cu^{II}$ d-d transition at 675 nm (Figure S15).

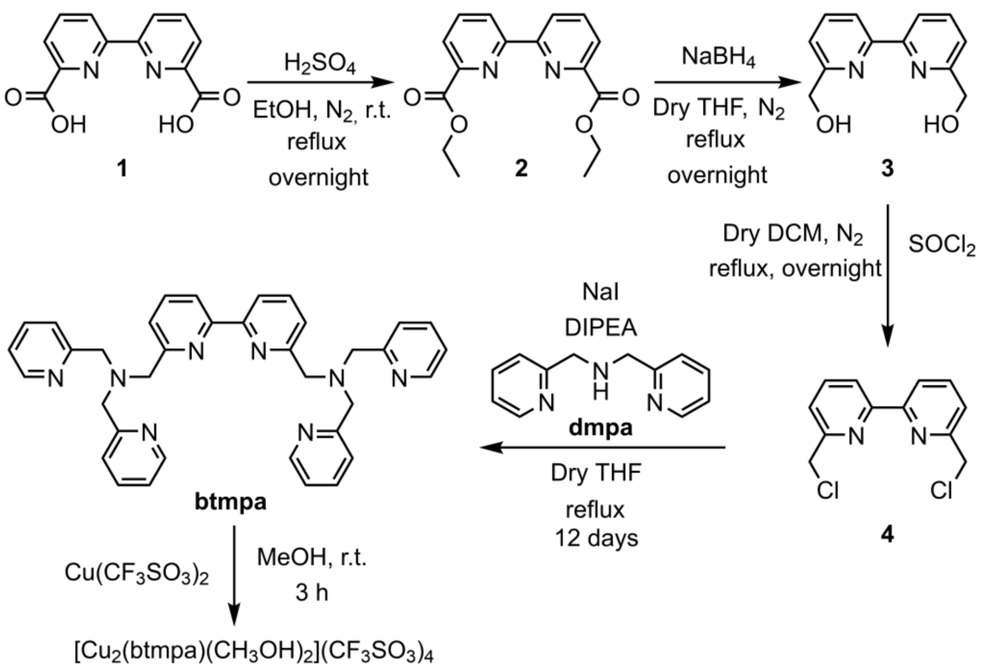


***Scheme S1**. Synthesis of btmpa and $[Cu_2(btmpa)(CH_3OH)_2](CF_3SO_3)_4$.*

## Synthesis of 6,6'-bis(hydroxymethyl)-2,2'-bipyridine (3)

**3** (Scheme S1) was synthesized with **2** as crude intermediate. **2** was synthesized following the procedure for synthesizing the methyl ester adapted from Forato *et al*.[3] ethanol (EtOH) instead of methanol (MeOH) was used to prepare the ethyl ester. 803.7 mg (3.29 mmol) of 2,2'-bipyridine-6,6'-carboxylic acid (ChemCruz, **1**) was loaded into a dry flask under inert atmosphere. 120 ml degassed EtOH (99.8%, Riedel-de Haën) was added to dissolve the compound. 16 ml of $H_2SO_4$ (98%, VWR) was added drop-wise over the course of 10 min. The mixture was set to reflux overnight. After cooling the mixture to 0 °C, it was slowly poured into 150 ml of saturated $NaHCO_3$ solution. The resulting mixture was extracted using 4 x 100 ml DCM (dichloromethane, Honeywell, >99.9%). The organic layers were collected, and the solvent was evaporated under reduced pressure resulting in a crude white solid which was characterized by $^1$H NMR and ESI MS. Besides peaks belonging to **2**, the monosubstituted by-product was found as well. This and other impurities were removed in the next step of the synthesis. ESI MS of **2** *m/z* (found (calculated)): 301.1 (301.3 [M + $H^+$]); 323.1 (323.3 [M + $Na^+$]). $^1$H NMR of **2** (300 MHz, $CDCl_3$) δ 8.88 (dd, 2H, $^3J$(H,H) = 7.9 Hz, $^4J$(H,H) = 1.1 Hz, Py-NCC-Py-C*H*), 8.21 (dd, 2H, $^3J$(H,H) = 7.8 Hz, $^4J$(H,H) = 1.1 Hz), 7.99 (t, 2H, $^3J$(H,H) = 7.8 Hz), *p*-Py-*H*), 4.53(q, 4H, $^3J$(H,H) = 7.1 Hz, $CH_3C\mathit{H_2}$); 1.49 (t, 6H, $^3J$(H,H) = 7.1 Hz, $C\mathit{H_3}CH_2$).

The crude product **2** was further used to synthesize **3** without further purification. **3** was synthesized following the procedure by Ganesan *et al*.[4] 1.1561 g of **2** was dissolved in 40 ml of dry, degassed THF (tetrahydrofuran, Sigma Aldrich) and loaded into a dry flask with inert atmosphere. The solution was further degassed using an $N_2$ flow for 30 min. 1.464 g (38.7 mmol) of $NaBH_4$ (Aldrich) was added to this solution. 9 ml of dry, degassed MeOH (Honeywell, >99.9%) was dropwise added by syringe, resulting in the formation of a large amount of gas. Once the gas production stopped, the mixture was set to reflux overnight. 150 ml of saturated $NH_4Cl$ solution, prepared using $NH_4Cl$ (Honeywell) and demineralized water, was added to the reaction mixture to neutralize the remaining $NaBH_4$. The resulting mixture was extracted using 3 x 100 ml EtOAc (ethyl acetate, VWR, Rectapur®). The organic layers were collected, and the solvent was evaporated under reduced pressure. 687.8 mg of product was obtained in the form of a white/yellow solid (3.18 mmol, 97% yield with respect to **1**). $^1$H NMR matches the values reported by Ganesan *et al*.[4] within 0.2 ppm. ESI MS *m/z* (found (calculated)): 217.0

(217.1 [M + H$^+$]); 239.0 (239.1 [M + Na$^+$]). $^1$H NMR (300 MHz, $CDCl_3$) δ 8.41 (dd, 2H, $^3J$(H,H) = 7.8 Hz, $^4J$(H,H) = 1.0 Hz, Py-NCC-Py-C*H*), δ 7.87 (t, 2H, $^3J$(H,H) = 7.8 Hz, *p*-Py-*H*), δ 7.52 (dd, 2H, $^3J$(H,H) = 7.8 Hz, $^4J$(H,H) = 1.0 Hz, Py-NCC-OH-C*H*), 4.77 (s, 4H, C*H$_2$*).

## Synthesis of 6,6'-bis(chloromethyl)-2,2'-bipyridine (4)

687.8 mg of **3** was dissolved in 60 ml of dry, degassed DCM (Sigma Aldrich) and loaded into a dry flask with inert atmosphere. The resulting solution was further degassed by purging with $N_2$ for 30 min. 1.2 ml of $SOCl_2$ (Acros Organics) was added dropwise, resulting in a turbid yellow solution. After stirring for 15 minutes the solution had turned clear and yellow. The mixture was left to stir under reflux overnight. After attaching a gas trap filled with 40 ml 10 M NaOH solution, 100 ml of water was carefully added to the reaction mixture to neutralize the remaining $SOCl_2$. The mixture was left stirring vigorously for 1 h to allow the $SOCl_2$ to neutralize completely. The reaction mixture was extracted using DCM (3 x 100 ml). The organic layers were collected, and the solvent was evaporated under reduced pressure resulting in a yellow solid. 643.8 mg of product was obtained (2.54 mmol, 80% yield). The recorded $^1$H NMR spectrum matches reported values with a maximum 0.05 ppm deviation.[5] MS *m/z* (found (calculated)): 252.9 (253.1 [M + H$^+$]). $^1$H NMR (300 MHz, $CDCl_3$) δ 8.41 (dd, 2H, $^3J$(H,H) = 7.9 Hz, $^4J$(H,H) = 1.0 Hz, Py-NCC-Py-C*H*), δ 7.87 (t, 2H, $^3J$(H,H) = 7.8 Hz, *p*-Py-*H*), 7.52 (dd, 2H, $^3J$(H,H) = 7.7 Hz, $^4J$(H,H) = 1.0 Hz, Py-NCC-Cl-C*H*), 4.77 (s, 4H, C*H$_2$*)

## Synthesis of 6,6’-bis(2,2’-dipicolylamine)-2,2’-bipyridine (btmpa)

### *Synthesis and initial purification*

465.1 mg of **4** and 16.4 mg of NaI were loaded into a dry flask attached to a Schlenk setup. The solids were degassed and subsequently dissolved in 80 ml Degassed, dry THF. 3.2 ml DIPEA (N,N-diisopropylethylamine, Sigma Aldrich) and 0.66 ml 2,2’-dipicolylamine (dmpa, Chemodex) were added to the solution. The resulting yellow solution was refluxed for 12 days, while the progress was checked by NMR. After 12 days, 200 ml of saturated $NaHCO_3$ solution was added followed by an extraction using 4 x 100 ml DCM. The organic layers were collected, and all volatiles were evaporated under reduced pressure. A silica column (Silica gel 40 – 63 µm, 60 Å, Screening Devices) using a gradient eluent of 0-10% MeOH in DCM was performed as a first purification. Column chromatography alone was not enough to remove unreacted dmpa since it has a very similar Rf value as btmpa and both compounds suffer from tailing and thus mixing. From several recrystallization attempts from 2 : 3 DCM : $Et_2O$ (diethyl ether, Honeywell), part of the crude product could be purified and btmpa was obtained as white crystals. 113.9 mg (0.197 mmol) of btmpa was obtained as first batch.

### *Purification with hexanoic anhydride*

As mentioned, unreacted dmpa is hard to remove from btmpa because the Rf values are very similar and both compounds tail slightly on alumina and largely on silica columns. A large difference in Rf would help to avoid mixing due to tailing of the compounds. Therefore, hexanoic anhydride (Sigma Aldrich) was used to change the Rf of dmpa. This method is based on the reaction of the secondary amine of dmpa with the anhydride to obtain an amide. The aliphatic tail of the amide makes the impurity more hydrophobic and, as a result, increases its Rf value with respect to that of btmpa. btmpa itself has no primary or secondary amines that can react with the anhydride. This way the compounds could be purified successfully. Specifically, 140 mg crude product containing btmpa, which was left after the first purification by column chromatography, was dissolved in 5 ml dry DCM (Sigma Aldrich), 135 µL DIPEA and 60 µL hexanoic anhydride (Sigma Aldrich) were added to the solution. The reaction mixture was stirred at room temperature for 2.5 hours. Next, the mixture was neutralized by adding 1 M HCl till the pH reached 7. The crude mixture was separated in two layers and the aqueous layer was extracted 3 times with DCM. All organic layers were combined, dried over $Na_2SO_4$ and filtered. All volatiles were removed under reduced pressure. For the purification, the product was dissolved in

DCM and loaded on an alumina column (Brockmann Type 1 Basic alumina). A gradient eluent was used from 0.2% triethylamine to 0.5% with additional 0.1% MeOH in DCM. The product fractions were washed with a saturated $NaHCO_3$ solution. Subsequently, this $NaHCO_3$ solution was extracted 3 times with DCM. All organic fractions were combined, dried over $Na_2SO_4$, and filtered before the volatiles were removed under reduced pressure. The obtained white solid was washed twice with pentane and once with $Et_2O$. After drying, 108 mg (0.187 mmol) of btmpa was obtained. $^1H$ NMR and MS confirmed the presence of btmpa and the absence of dmpa and hexanoic amide. The $^1H$ NMR spectra matches reported spectra.[6] The total yield of pure product is 241.9 mg (0.418 mmol, 23%). ESI MS *m/z* (found (calculated)): 601.5 (601.28 [M + $Na^+$]); 579.5 (579.3, [M + $H^+$]); 290.3 (290.15, [M + 2 $H^+$]). $^1H$ NMR (300 MHz, $CDCl_3$) δ 8.53 (dt, 4H, $^3J$(H,H) = 4.9 Hz, $^4J$(H,H) = 1.4 Hz, *o*-Py-*H*), 8.30 (dd, 2H $^3J$(H,H) = 7.8 Hz, $^4J$(H,H) = 1.1 Hz, Py-NCC-Py-C*H*), 7.77 (t, 2H, $^3J$(H,H) = 7.7 Hz, *p*-biPy-*H*), 7.72 – 7.58 (m, 8H, *p*-Py-*H* and *m*-Py-*H*), 7.54 (dd, 2H, $^3J$(H,H) = 7.7 Hz, $^4J$(H,H) = 1.1 Hz, biPy-NCCN-C*H*), 7.14 (m, 4H, Py-$NCHCH_2N$-C*H* ), 3.95 (overlapping singlets, 12H, C$H_2$).

In total, both batches (113.9 and 108 mg) gave a total yield of 221.9 mg (0.383 mmol, 21%).

## Synthesis of $[Cu_2(btmpa)(CH_3OH)_2](CF_3SO_3)_4$ ($Cu_2$(btmpa))

108 mg of **btmpa** was dissolved in 5 ml MeOH yielding a yellow solution. Next, 136 mg of Cu(II)$(CF_3SO_3)_2$ (Alfa Aesar) was dissolved in 1 ml MeOH and added to the ligand solution. The solution turned from green to dark blue upon addition. The mixture was stirred for 3 hours at room temperature after which the solvent was removed under reduced pressure. The remaining solid residue was redissolved in 2 ml MeOH and $Et_2O$ was slowly added by vapor diffusion at 4 °C temperature. The obtained blue crystals were filtered and washed with cold $Et_2O$ and dried. After repeating the crystallization, 162 mg (0.118 mmol, 63%) blue crystals of the complex were obtained. The calculated (%) elemental analysis ratio (%) for $[Cu_2(btmpa)(MeOH)_2](OTf)_4$ ($C_{42}H_{42}Cu_2N_8O_{14}S_4$) + 2.5 $H_2O$ : C 35.75, H 3.36, N 7.94; found: C 35.48, H 3.10, N 7.78. UV-vis $\lambda_{max}$: 228 nm, 675 nm (0.15 mM in water); 632 nm, 816 nm (2.0 mM in MeCN). EPR 0.6 mM in $H_2O$: $g_{||}$ = 2.21, $g_{\perp}$ = 2.08; phosphate buffer: $g_{||}$ = 2.23, $g_{\perp}$ = 2.06; in dimethyl formamide: $g_{||}$ = 2.23 ($A_{Cu}$ = 500 Hz), $g_{\perp}$ = 2.06. (Figure S1).

## XPS

The GC electrodes used for measuring XPS were custom-made disks with 5.0 mm diameter. Before the measurement, they were mechanically polished as described below. After the electrochemical measurement, the electrode was removed from the setup and rinsed copiously with Milli-Q grade Ultrapure water. Next, the electrodes were dried under a soft stream of $N_2$ and shipped in air to TU Darmstadt, where they were handled, mounted, and introduced to the XPS load lock under ambient air conditions.

XPS measurements have been performed on a PHI Versaprobe 5000 using monochromatic Al Kα (hν = 1486.6 eV) radiation, a power of 50 W and a round measurement spot with a diameter of 200 µm. Region scans (C 1s, N 1s, Cu 2p shown here) have been recorded with a pass energy of 23.5 eV and a step size of 0.1 eV and the survey spectra with 187.85 eV pass energy and 1.0 eV step size at a constant analyzer angle of 45°. No charge compensation was applied to samples on GC electrodes. For the reference powder samples mounted on conductive carbon tape, charge neutralization by electron and $Ar^+$ ion flooding was applied. Qualitative analyses of the XPS spectra was performed in CasaXPS software, version 2.3.22. Binding energy calibration was done by setting the binding energy of the C 1s component corresponding to $sp^2$ carbon to 284.5 eV.

## General electrochemistry

For all aqueous solutions, all experiments and for cleaning of glassware Milli-Q grade Ultrapure water (>18.2 MΩ cm resistivity) was used unless mentioned otherwise. The pH 7 electrolyte was prepared

with $NaH_2PO_4$ (Merck Suprapur ©, 99.99%) and $Na_2HPO_4$ (Fluka Traceselect© 99.995%) with 0.1 M phosphate strength. Electrochemical experiments were performed with a three-electrode setup in a custom-made, single-compartment glass cell. For EQCM, RRDE and bulk electrolysis, special cells were used that are described separately. Autolab PGSTAT 12, 204, 128N and IVIUM CompactStat potentiostats were used in combination with NOVA 2.1 or IVIUM software. All glassware used for electrochemistry was cleaned by boiling in and copiously rinsing the glassware with water prior to each experiment. Periodically and before each RRDE measurement, the glassware was cleaned by immersing the glassware in a 1 g/l $KMnO_4$ solution in 0.5 M $H_2SO_4$ (Sigma, reagent grade) overnight. Afterwards, the glassware was rinsed 5–10 times with water. To re-oxidize any $MnO_2$ traces, water, a few drops of $H_2O_2$ (Merck Emprove, 35%) and $H_2SO_4$ (Merck) were added. Finally, the glassware was rinsed 5-10 times and boiled in water for a total of three times

All electrolyte solutions were purged by argon (Linde, Ar 5.0) prior to each experiment for at least 30 minutes and the cell was kept under a flow of argon during the experiment. For experiments under $O_2$ (Linde, $O_2$ 5.0), the electrolyte was purged with $O_2$ for at least 10 minutes prior to the measurement and was purged continuously during RRDE measurements.

In all cases, the reference electrode was the reversible hydrogen electrode by utilizing a platinum mesh in $H_2$ (Linde, $H_2$ 5.0) saturated electrolyte that is operated at the same pH as the working electrode or by using a HydroFlex (Gaskatel). The cell and reference electrode are connected via a Luggin capillary. The counter electrode was a large surface area gold wire that was flame annealed prior to use. The working electrode was glassy carbon (GC, Metrohm, 0.07 $cm^2$) encapsulated in PEEK (polyether ether ketone). The GC electrode was polished before each measurement. Either manual or mechanical polish methods were used. Manual polishing was applied with 1.0, 0.3 and 0.05 micron alumina slurry (Buehler) on MicroCloth (Buehler) polishing cloths for 2 minutes followed by rinsing and sonicating the electrode in water for 10 minutes. Mechanical polishing was applied with a Labopol-20 polishing machine on Dur type polishing cloths with 1.0 micron diamond and 0.04 micron silica suspensions for 1 minute (Struers). After the diamond polishing, the electrode was rinsed with water and 2-propanol to remove the oily substances from the slurry. The silica polish was followed by a rinse with water. After that, the electrode was sonicated in water for 10 minutes.

## EQCM

EQCM experiments were performed with an Autolab gold EQCM electrode (0.35 $cm^2$) that consist of a 200 nm thick gold layer deposited on a quartz crystal. An adjusted Autolab PEEK EQCM cell was used that was able to contain up to 5 ml of electrolyte.

## RRDE

RRDE experiments were performed in a three electrode setup with custom-made two-compartment cells that could separate the working electrodes from the counter electrode via a water permeable glass frit. A glassy carbon disk (0.196 $cm^2$) surrounded by a platinum ring in a ChangeDisk configuration was used in a Pine MSR rotator as supplied by Pine Instruments. The GC disk and Pt ring electrodes were, prior to each measurement, separately polished. Both were either manually or mechanically polished. For the GC disk, the same procedure for the PEEK encapsulated disk was used. For Pt, the manual polish was performed with 1.0 and 0.3 micron alumina for 30 seconds and 0.05 micron alumina for 1 minute after which the electrode was sonicated for 10 minutes in water. The mechanical polish was equal to the treatment for the GC disk. When applicable, the Pt ring was further electropolished by performing CV in 0.1 M phosphate buffer of pH 7 for 50 scans at a 500 mV/s scan rate between 1.7 and –0.1 V while the shaft was rotated at 1600 rpm.

## Bulk electrolysis and Faradaic efficiency

The Faradaic efficiency was determined by bulk electrolysis in combination with $H_2O_2$ enzyme based photometric determination of the bulk $H_2O_2$ concentration with the Reflectoquant® system (Merck) using test strips for 0.2 to 20 mg/l $H_2O_2$ (Merck). The bulk electrolysis was performed in a custom-made glass cell with glass-frit separated compartments for the reference, work and counter electrode. A HydroFlex (Gaskatel) reference electrode was used as RHE reference. The counter electrode was a high surface area gold wire. The work electrode was a custom-made, mechanically polished GC electrode (0.196 $cm^2$) in a rotating disk setup from Pine. The electrode was continuously rotated at 1600 rpm. The initial volume of the 0.15 mM **$Cu_2$(btmpa)** solution was 33.6 ml. A circa 1.5 ml aliquot was taken for each $H_2O_2$ measurement and weighed to correct for the decrease in volume of the bulk solution during the measurement. Each aliquot was tested at least twice with the test strips immediately after removing it from the solution. The products of $H_2O_2$ dissociation by a peroxidase react with an organic dye on the test strip. The intensity of the color that arises can be used to quantify $H_2O_2$. The theoretical charge required to obtain the measured concentration difference of $H_2O_2$ in the time between two measurements was calculated. The actual charge that passed in the same time window was obtained by integration of the current over time. Thereby, the Faradaic efficiency could be calculated.

# EPR and SQUID of $Cu_2$(btmpa)

The electron paramagnetic resonance (EPR) spectra of **$Cu_2$(btmpa)** were measured in water, 0.1 M phosphate buffer and dimethyl formamide (Figure S1). In water and phosphate buffer, broad peaks were observed and two g values could be extracted from the simulation: $g_{||}$ = 2.21 and $g_{\perp}$ = 2.08 for water and $g_{||}$ = 2.23, $g_{\perp}$ = 2.06 for phosphate buffer though the latter simulation was less accurate. The spectra and g values are very similar but minor discrepancies suggest that phosphate coordination slightly changes the geometry around the copper cores though pH effects are not excluded. In dimethyl formamide, a clear splitting pattern arising from coupling to the copper core was observed. The g values of dimethyl formamide are $g_{||}$ = 2.23 ($A_{Cu}$ = 500 Hz) and $g_{\perp}$ = 2.06, equal to the phosphate buffer sample. This might indicate that both dimethyl formamide and phosphate have a similar influence on the geometry. Because $g_{||} > g_{\perp}$, the single occupied molecular orbital (SOMO) is the $d_{x^2-y^2}$ orbital since there is significant orbital mixing in the $z$-direction ($g_{||} = g_z$). This is the case in, for example, an elongated (distorted) octahedron.[7] Hence, the geometry of **$Cu_2$(btmpa)** in aqueous and dimethyl formamide solutions is in close resemblance to the previously published crystal structures.[6] The obtained g tensors are clearly different from the mononuclear **Cu(tmpa)** complex ($g_{||}$ = 2.00, $g_{\perp}$ = 2.19) indicating a significant difference in geometry.[8] In the case of **Cu(tmpa)**, $g_{||} < g_{\perp}$, pointing to $d_{z^2}$ as SOMO and a trigonal bipyramidal geometry of the complex.[7] Overall, it seems that both copper centra of **$Cu_2$(btmpa)** can be seen as independent and produce the same EPR signal. There is no antiferromagnetic coupling as was also observed with SQUID (superconducting quantum interference device, Figure S3). Interestingly, the g value for both copper centra was found to be 1.85 from the powder SQUID spectra which indicates that they are identical but have a lower value than determined from the frozen solution EPR spectra. It has to be noted that g values from EPR are generally more accurate than g values obtained from fitted SQUID data. From the SQUID data it can also be concluded that there is a small ferromagnetic coupling between the two copper centra of 34 $cm^{-1}$.

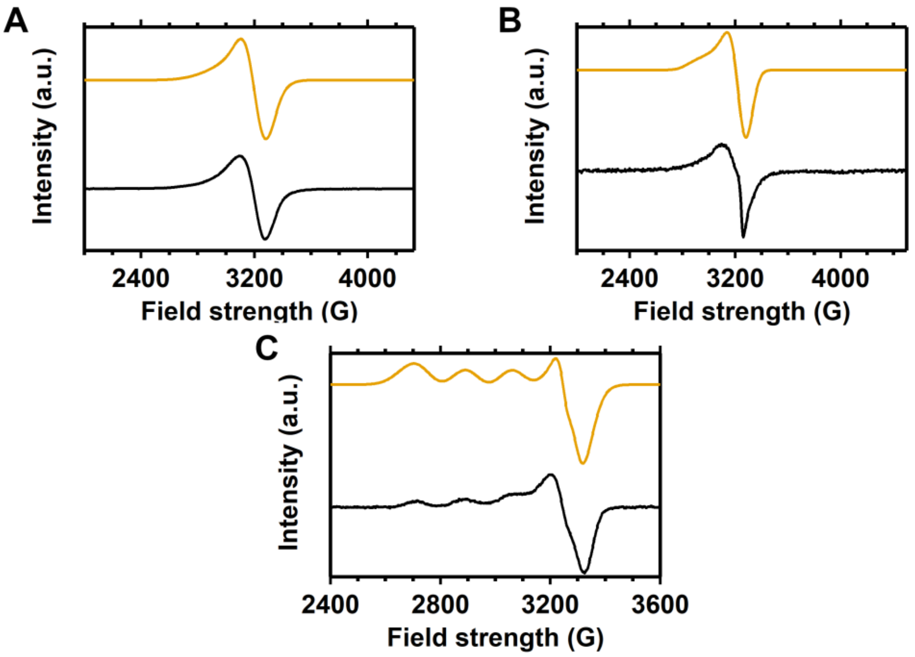


***Figure S1.*** *EPR spectra (black lines) of 0.6 mM **$Cu_2$(btmpa)** in water (A, 9.342 GHz), phosphate buffer (B, 9.344 GHz) and dimethyl formamide (C, 9.352 GHz). The simulated spectra are shown in orange. Spectra were obtained at 77 K with frozen solutions.*

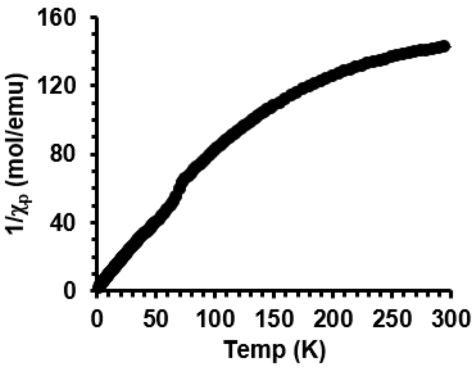


***Figure S2.*** *SQUID spectrum of* ***$Cu_2$(btmpa)*** *powder.*

# Additional electrochemical data of $Cu_2$(btmpa)

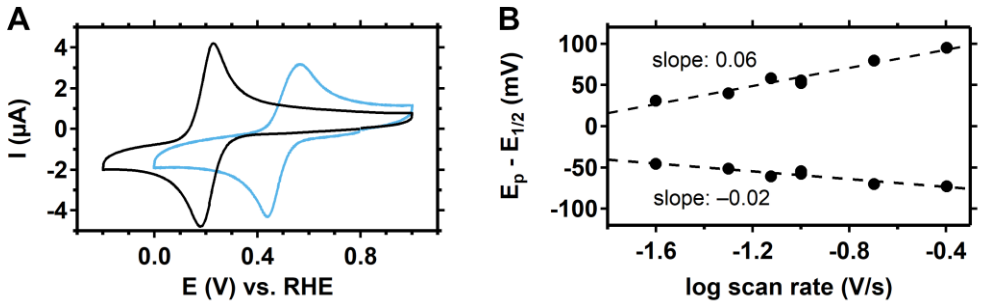


***Figure S3**. (A) Cyclic voltammogram of a 0.15 mM **$Cu_2$(btmpa)** solution (blue) and a 0.3 mM **Cu(tmpa)**[9] solution (black) under an argon atmosphere in a 0.1 M phosphate buffer of pH 7 with a scan rate of 100 mV/s. (B) Laviron plot of the cathodic and anodic peak positions of **$Cu_2$(btmpa)** and the slopes of linear fits.*

Using the equation,

$$\psi = \frac{k_{ET}\sqrt{\frac{D_{ox}}{D_{red}}}}{\sqrt{\frac{\pi D_{ox} n F v}{RT}}}$$

a $k_{ET}$ of 0.09 cm $s^{-1}$ was calculated for **$Cu_2$(btmpa)**. Herein ψ is a dimensionless parameter that is related to $\Delta E_p$ and taken from reference [10], $D_{ox}$ and $D_{red}$ are diffusion constants of **$Cu_2$(btmpa)** obtained by plotting the peak current versus the square root of the scan rate ($D_{red}$ = $1.6 \times 10^{-6}$ $cm^2$ $s^{-1}$; $D_{ox}$ = $9.1 \times 10^{-7}$ $cm^2$ $s^{-1}$).

Electrochemical Quartz Crystal Microbalance experiments

EQCM is an *in-situ* technique that probes the mass changes of the work electrode by monitoring the change in oscillation frequency of the quartz crystal on which the work electrode resides.[11] In EQCM, a negative difference in frequency corresponds to an increase of the mass of the electrode. This technique visualizes any permanent deposit on the electrode as is sometimes formed by molecular complexes.[11] Specifically, a gold electrode on such a quartz crystal was used for this purpose (Figure 1 (2nd scan) and Figure S4 (entire experiment). The relative frequency of the oscillation decreases as soon as the complex is reduced electrochemically in the absence of $O_2$ starting at 0.5 V while scanning negative. In the positive scan, the complex is re-oxidized above 0.5 V which is accompanied by an increase of the frequency back to the starting frequency. Hence, the $Cu^{II/I}$ redox couple triggers a reversible change in mass of the electrode. This illustrates that no permanent deposit is formed. The origin of the reversible adsorption might be due to a change in solubility of the complex when changing the charge from 4+ to 2+. The less-charged complex could subsequently adsorb on the electrode more readily.

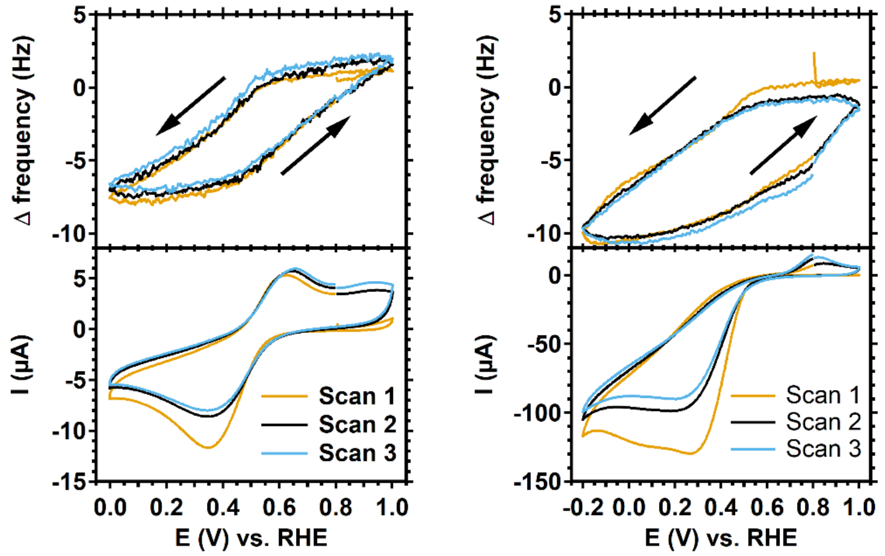


***Figure S4.*** *Electrochemical quartz crystal microbalance measurement with a gold work electrode of 0.15 mM* ***$Cu_2$(btmpa)*** *in 0.1 M phosphate buffer of pH 7 under Argon (left) and under $O_2$ (right). The bottom panel shows CV cycles at 50 mV/s scan rate under argon atmosphere. The first scan under Argon deviates because not all oxygen was completely removed. The top panel shows the relative frequency of the quartz crystal and its response with respect to the applied potential.*

# Additional RRDE data of $O_2$ reduction by $Cu_2$(btmpa)

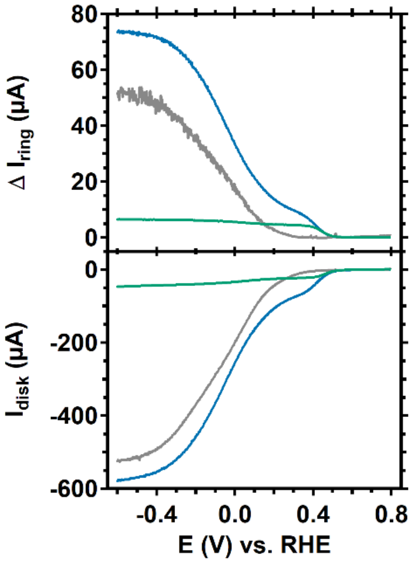


***Figure S5.*** *Full potential window of Figure 2. Linear sweep voltammograms with a rotating ring disk electrode setup of 0.15 mM **$Cu_2$(btmpa)** under argon (green) and $O_2$ atmosphere (blue). The cyclic voltammogram of the GC disk (bottom panel) and the current response of the Pt ring (top panel) are shown. The grey line represents the GC disk in catalyst-free, $O_2$ purged electrolyte. The voltammograms were recorded at 50 mV/s in a 0.1 M phosphate buffer of pH 7. A rotation rate of 1600 rpm and a Pt ring potential of 1.2 V were applied.*

Shown in Figure 2 and Figure S5 is the electrochemical oxygen reduction reaction (ORR) of **$Cu_2$(btmpa)** studied by cyclic voltammetry (CV) with a rotating ring disk electrode (RRDE) setup. This setup allows for controlled mass transport due to continuous rotation of the electrode resulting in a laminar flow of $O_2$ purged electrolyte towards the electrode. Furthermore, a Pt ring around the work electrode can be used as electrochemical sensor for the oxidation of $H_2O_2$ by applying a potential of 1.2 V. The onset for ORR for the glassy carbon (GC) work electrode itself is at *circa* 0.35 V *versus* RHE under our conditions (Figure 2) which generally selectively performs the 2 electron reduction of $O_2$ to $H_2O_2$.[12] Indeed, the production of $H_2O_2$ could be derived from the increase in ring current as soon as $O_2$ was reduced (Figure 2). When **$Cu_2$(btmpa)** was present in solution under an argon atmosphere, the onset for complex reduction was at 0.50 V. In addition, a ring current was observed which corresponds to the re-oxidation of the **$Cu^I_2$(btmpa)** at 1.2 V. When the solution was saturated with $O_2$, the onset lies at 0.50 V as well. However, the disk current exceeded the current in absence of $O_2$ pointing to the fact that catalytic $O_2$ reduction took place. Likewise, the ring current exceeded the current in absence of $O_2$ indicating that $H_2O_2$ is formed. In the potential window between 0.50 and 0.35 V, the GC electrode itself is not active for $O_2$ reduction. Hence, **$Cu_2$(btmpa)** must perform $O_2$ reduction and produce $H_2O_2$. Below 0.35 V, the disk (and ring) current increased significantly because GC reduces $O_2$ as well in this potential window and a diffusion limited current was reached at –0.6 V (Figure S5). In this potential window, $O_2$ reduction is mostly performed by GC. Also, the diffusion limited current in presence of **$Cu_2$(btmpa)** is reached at the same potential as in the absence of **$Cu_2$(btmpa)**. **$Cu_2$(btmpa)** clearly reduces $O_2$ slower as compared to the mononuclear complex **Cu(tmpa)** as the latter reaches a diffusion limited current at 0.2 V.[9]

## $H_2O_2$ selectivity of $Cu_2$(btmpa)

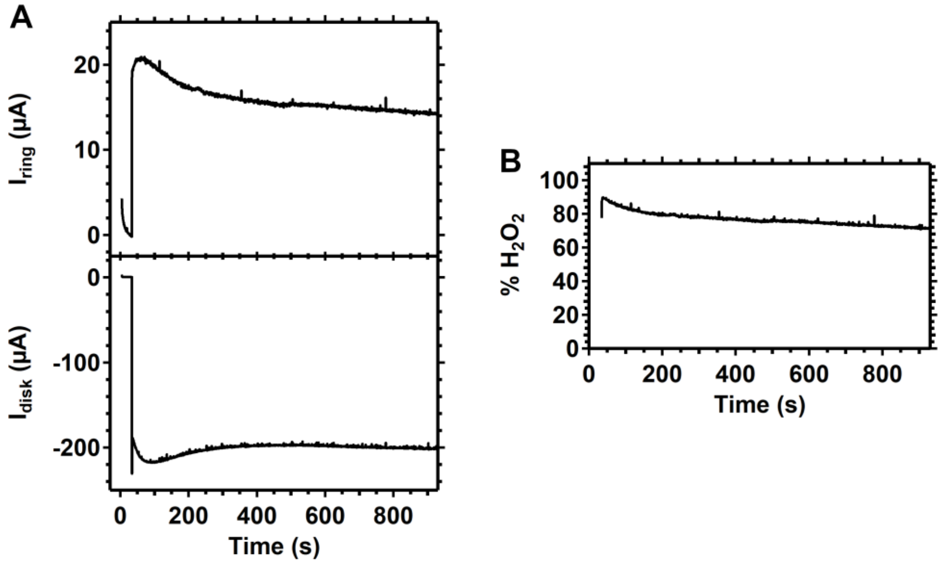


***Figure S6.*** *Chronoamperometry with a RRDE setup (A) of $O_2$ reduction in presence of* ***$Cu_2$(btmpa)*** *at 0.2 V disk potential (bottom panel) and corresponding Pt ring current (top panel). The calculated $H_2O_2$ selectivity is shown in B. A 0.1 M phosphate buffer at pH 7 with 0.15 mM catalyst was used. The GC disk was rotated at 1600 rpm. The Pt ring was kept at 1.2 V. The collection efficiency was determined at 17.5% determined by a 3 minute amperometry measurement at –0.3 V in catalyst-free electrolyte.*

## Collection efficiency of $H_2O_2$ on a Pt ring electrode in a RRDE setup

For reliable rotating ring disk electrode (RRDE) measurements, the collection efficiency has to be stable throughout the measurement. That way, the percentage of $H_2O_2$ and the electron transfer number ($n$) can be determined using equations S1 and S2 respectively where $N_{H_2O_2}$ is the collection efficiency for $H_2O_2$. Usually, this collection efficiency can be determined with a one-electron redox couple such as the $[Fe^{II}CN_6]^{4-}/[Fe^{III}CN_6]^{3-}$ and equation S2 where $N_{CE}$ is the collection efficiency. However, $H_2O_2$ oxidation on Pt is not always diffusion limited. As a result, the $H_2O_2$ collection efficiency ($N_{H_2O_2}$) can be different from the $N_{CE}$ determined with the $Fe^{II/III}$ redox couple.[9] Phosphate buffer and $PtO_x$ formation at 1.2 V can significantly influence the amount of active sites thereby changing the potential where diffusion limited $H_2O_2$ oxidation is obtained on a Pt ring.[13] To study what factors influence $N_{H_2O_2}$, several chronoamperometry measurements were performed with the RRDE setup using a GC disk as work electrode in $O_2$ purged 0.1 M phosphate buffer of pH 7. The GC electrode is a 100% selective catalyst at moderate potentials in the freshly polished state.[12]

$$\%H_2O_2 = \frac{2 \times (\frac{i_{ring}}{N_{H_2O_2}})}{i_{disk} + (\frac{i_{ring}}{N_{H_2O_2}})} \times 100\% \qquad \textbf{Eq. S1}$$

$$n = \frac{4 \times i_{disk}}{i_{disk} + (\frac{i_{ring}}{N_{H_2O_2}})} \qquad \textbf{Eq. S2}$$

$$N_{CE} = \frac{|i_{ring}|}{|i_{disk}|} \qquad \textbf{Eq. S3}$$

To start, we studied the influence of the disk potential (Figure S7). The ring and disk currents were stable at disk potentials (0.1, 0.0, and –0.1 V) where low current is obtained (Figure S7A). The corresponding collection efficiencies are lower than the theoretical value of 24% for this specific setup (Figure S7B). The maximum collection efficiency can be achieved by not only mechanically polishing the Pt ring, but also electropolishing the ring to remove any poisoning substances from the Pt surface. However, we found that such a clean Pt surface is very susceptible for (re-)poisoning and therefore the collection efficiency will drop quickly over the course of the experiment. When merely mechanical polish is applied, the collection efficiency shows a minor incline over the course of the 10 minute experiment (Figure S7B) and is therefore more useful. Nevertheless, only a low amount of $H_2O_2$ is produced at these potentials since the disk current is low. The disk produces up to 6 times more current at –0.6 V. Interestingly, the disk current increases over the course of 10 minutes whereas the ring current decreases at that potential. As the ratio ring to disk current decreases rapidly, the calculated collection efficiency (Figure S7B) decreases from 14 to 8% over the course of 10 minutes at –0.6 V disk potential. Two explanations are possible. First, the selectivity of $O_2$ to $H_2O_2$ might not be 100% at this potential but instead an increasing part of the current might be attributed to the over-reduction of $H_2O_2$ to $H_2O$. The second explanation is that the Pt ring surface is affected by $H_2O_2$ and therefore less able to oxidize $H_2O_2$.

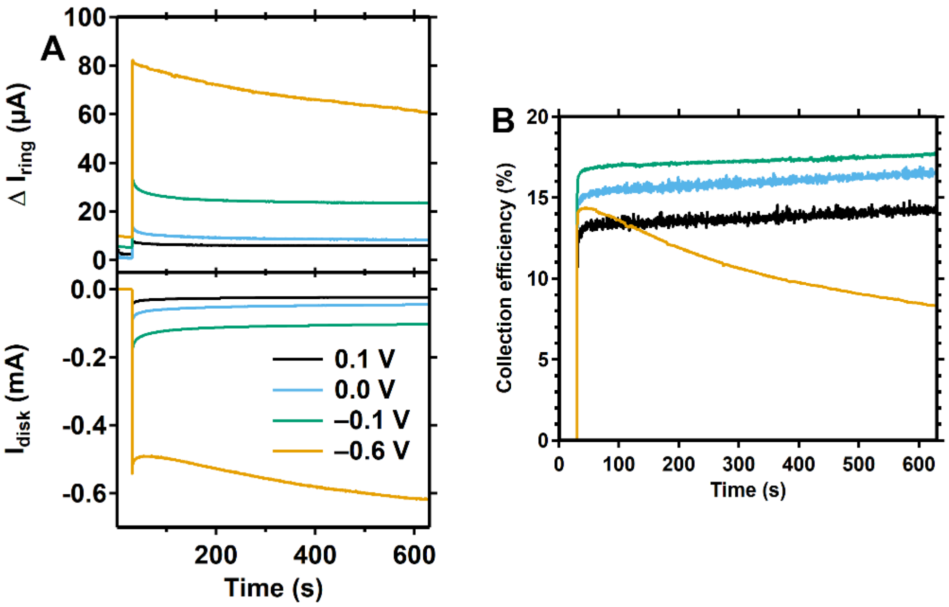


***Figure S7.*** *RRDE Chronoamperometry of $O_2$ reduction by a GC disk at various potentials (A, bottom panel), the response of the Pt ring (A, top panel), and the calculated collection efficiency of the ring for $H_2O_2$ (B). The ring was kept at 1.2 V. The disk was rotated at 1600 rpm. All electrodes were polished before the measurement. $O_2$ purged 0.1 M phosphate buffer of pH 7 was used.*

To study whether the Pt surface is affected by $H_2O_2$, we performed two RRDE amperometry measurements at 0.0 V for 10 minutes. First, a freshly polished GC and Pt ring were used (Figure S8). After the first 0.0 V measurement, amperometry at –0.6 V was performed for 10 minutes in order to expose the Pt ring to a large amount of $H_2O_2$. Finally, another 0.0 V amperogram was recorded for 10 minutes. The disk current at 0.0 V for the last measurement is lower than it the freshly polished state, but more stable at –45 µA. The current of the freshly polished GC decreases to circa –50 µA after 10 minutes. On the other hand, the ring current has significantly dropped from circa 10 µA to 5 µA in the 0.0 V measurement after the –0.6 V measurement. Moreover, the ring to disk ratio, and consequently the calculated collection efficiency, drops significantly over the 10 minute experiment from 15 to 9%. In conclusion, the Pt ring is somehow affected and cannot consistently oxidize $H_2O_2$ after being exposed to larger amounts of $H_2O_2$. Indeed, earlier reports have shown that $H_2O_2$ inhibits $H_2O_2$ oxidation by Pt at high (>1 mM) $H_2O_2$ concentration as the process is a mix of diffusion and kinetical parameters.[14] Therefore, the application of RRDE for the detection of $H_2O_2$ remains limited to situations where a low amount of $H_2O_2$ is produced and/or a qualitative one where only the detection is important. Quantification of $H_2O_2$ in situations with larger amounts of $H_2O_2$ is limited under these conditions.

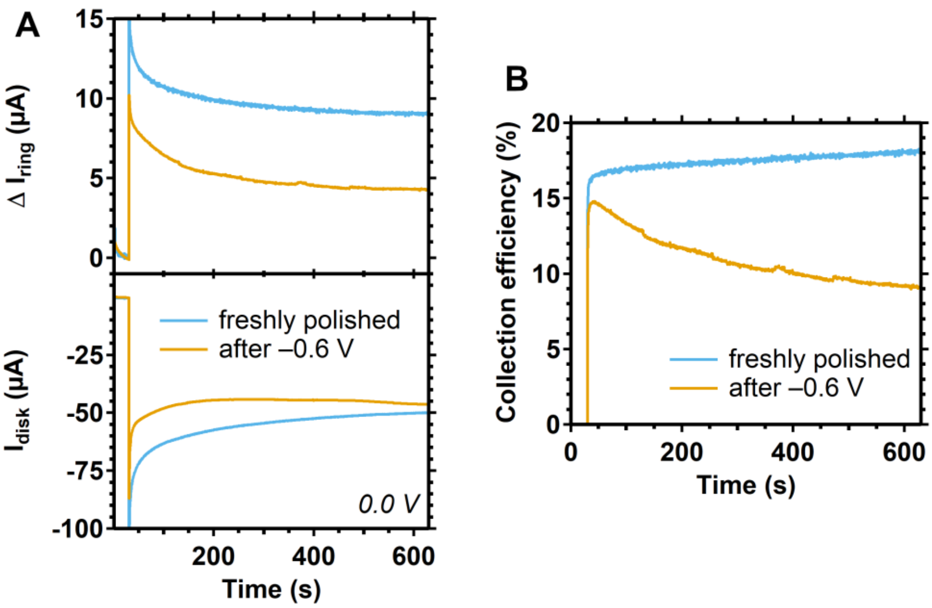


***Figure S8.*** *RRDE chronoamperometry of $O_2$ reduction at 0.0 V for 10 minutes (A, bottom panel), the Pt ring response (A, top panel) with a freshly polished GC an Pt ring (blue) and after an experiment that produced high amounts of $H_2O_2$ (orange). The corresponding calculated collection efficiencies (or ring to disk ratios) are shown in B. Prior to the yellow measurement, an amperogram at –0.6 V was recorded for 10 minutes. The ring was kept at 1.2 V. The same conditions as in Figure S7 were used.*

# Electrochemical $H_2O_2$ reduction by $Cu_2$(btmpa)

Over-reduction of $H_2O_2$ to $H_2O$ is a possibility that could lower the $H_2O_2$ selectivity. Therefore, $H_2O_2$ reduction by **$Cu_2$(btmpa)** under argon atmosphere was studied by CV (Figure S9). The reduction of different concentrations of $H_2O_2$ was studied under rotating and non-rotating conditions. Indeed, there is reducing current observed indicating that $H_2O_2$ is reduced by the complex. A positive order in $H_2O_2$ is expected for the rate determining step because the reducing current increases with the $H_2O_2$ concentration. Interestingly, no peak current under non-rotating and no diffusion limited current under rotating conditions could be obtained even at high concentrations of $H_2O_2$. Only at 1.2 mM, a peak current and limiting current seem to be reached. However, these disk currents of 50 µA are low and not close to 200 µA which is the diffusion limited current for $H_2O_2$ reduction under these conditions.[9] Overall, the reduction of $H_2O_2$ by **$Cu_2$(btmpa)** is a slow, kinetically limited reaction. Therefore, $O_2$ reduction to $H_2O_2$ by **$Cu_2$(btmpa)** can reach a high selectivity in this potential window.

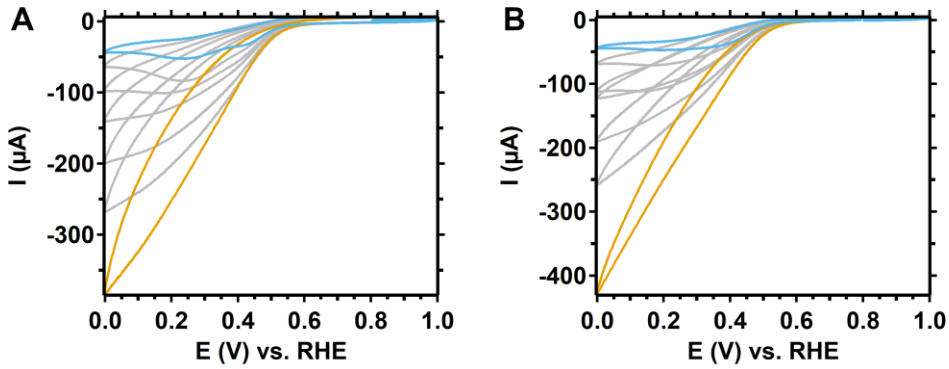


***Figure S9.*** *Cyclic voltammograms of $H_2O_2$ reduction by 0.15 mM **$Cu_2$(btmpa)** under argon atmosphere with a non-rotating (A) and rotating (B, 1600 rpm) GC electrode (0.196 $cm^2$). $H_2O_2$ concentrations of 1.2 (blue), 2.1, 4.2, 8.4, 14.1, 25.2, and 47.4 mM (orange) were tested in 0.1 M phosphate buffer of pH 7. Scan rates of 100 mV/s (A) and 50 mV/s (B) were used.*

# Desorption of $Cu_2$(btmpa) and copper deposition

Our experiments show two types of deposits. During early stages of chronoamperometry we see an increase in current, while good Faradaic Efficiencies are maintained. The observed currents are not directly affected by the first stripping experiments (at e.g. 30 minutes). Over time the Faradaic efficiency drops and signs of copper plating are observed. This deposit is affected by the stripping intervals. The currents revert to the original levels, and the Faradaic Efficiency restores.

To further set these materials apart we have conducted the following long term amperometry experiments and additional rinsing tests:

(a) Amperometery (0.0 V vs RHE) in the presence of 0.15 mM **$Cu_2$(bmpta)** for 7 hours (Figures 3 and S11).
(b) Amperometry (0.0 V vs RHE) in the presence of 0.15 mM **$Cu_2$(btmpa)** for 7 hours, while maintaining stripping intervals (0.8 V vs RHE) every 20 minutes (Figures 3 and S11).
(c) Amperometry (0.0 V vs RHE) in the presence of 0.15 mM **$Cu_2$(btmap)** for 30 minutes, followed by rinsing and subsequent amperometry (0.0 V vs RHE) for 5 hours in a blank electrolyte solution that does not contain any **$Cu_2$(btmpa)** (Figure S11).

During the first half hour of amperometry at 0.0 V a significant increase in current is observed in all cases. Moreover, in the first 2 hours of amperometry with 0.8 V intervals (b), the magnitude of the current at the end of each cycle is regained at the start of the next cycle after the interval (Figure 3). Apparently, the deposit causing this increase in current cannot be simply removed by applying 0.8 V and thus is different from $^{dep}$Cu. On the other hand, an electrode that was held in a **$Cu_2$(btmpa)** solution only for the first half hour and subsequently rinsed with water did not retain its activity (c). When this electrode was put in catalyst-free electrolyte after rinsing to compare the activity with a polished GC electrode, the current drops significantly to –0.08 mA and is equal the current of the polished GC (Figure S11). This in in contrast to what happens when the electrode stays in catalyst solution (Figure 3). In that case (a), the current remains –0.37 mA even after briefly applying a potential of 0.8 V. The rinsing of the electrode is the extra step that removed most of the deposit on the electrode. When the rinsed electrode was tested for an additional 5 hours (c), the current steadily increased to –0.27 mA. It did not reach the level of an electrode that permanently resides in a **$Cu_2$(btmpa)** solution (–0.83 mA, (a)) but it did follow a similar current profile. Also, the polished GC does not show this behavior (Figure S11). These results seem to point to a small residue of **$Cu_2$(btmpa)** still residing on the electrode even after the rinse. Although the EQCM data show that the potential-dependent adsorption is reversible on gold electrodes, the carbon-based GC electrode might have a stronger affinity with **$Cu_2$(btmpa)**. Especially in the first half hour of amperometry, catalyst accumulation on the electrode enhances the number of active sites. At 0 V, **$Cu_2$(btmpa)** is still significantly kinetically limited. In this case, an increase in active sites would indeed increase the catalytic current. The 0.8 V intervals do not lead to significant desorption of **$Cu_2$(btmpa)** perhaps aided by the continuous supply of fresh catalyst by the rotation of the electrode. Rinsing with water on the other hand, did remove most catalyst. The small residue that would still be present was then subjected to 0.0 V for 5 hours. In that time window, this residue can degrade to $^{dep}$Cu which explains why the current steadily increases more than that of a polished GC (Figure S11). Indeed, after applying a short 0.8 V interval, the current significantly decreased to –0.15 mA which indicated the stripping of $^{dep}$Cu (Figure S11).

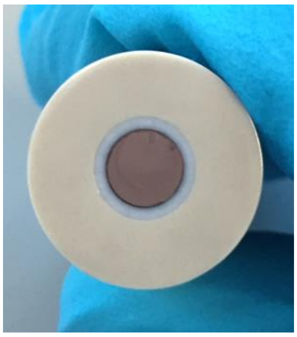

***Figure S10.*** *Picture of the copper-colored deposit (*$^{dep}$*Cu) on a GC electrode after 7 hours of amperometry at 0.0 V in presence of* ***$Cu_2$(btmpa)****.*

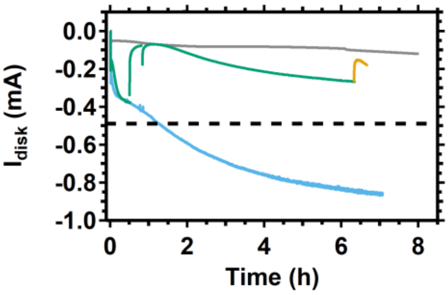


***Figure S11.*** *Rotating (1600 rpm) disk electrode experiment at 0.0 V of a glassy carbon electrode in* $O_2$ *saturated electrolyte in absence (grey) or in presence (blue) of* ***$Cu_2$(btmpa)****. The green trace was in presence of* ***$Cu_2$(btmpa)*** *for 30 minutes after which the electrode was rinsed and tested in a catalyst-free electrolyte. The orange trace is a 20 minute measurement recorded after the electrode (green trace) was briefly held at 0.8 V. 0.1 M phosphate buffer of pH 7 was used. Catalyst concentration was 0.15 mM.*

# Intercepting $H_2O_2$ during long term chronoamperometry

After more than two hours of chronoamperometry we observed that the Faradaic Efficiency started to drop, which we attribute to the buildup of hydrogen peroxide in the reaction mixture. To support this hypothesis we carried out an additional experiment wherein we set the Pt ring to continuously reoxidize the hydrogen peroxide formed during the experiment, leading to a significant longer catalyst lifetime.

In this experiment we performed amperometry at 0.0 V in 20 minute intervals for a period of 8.5 hours. In the 10 second intervals, potentials of 0.55 V at the Pt ring to reduce accumulated $PtO_x$ and 0.8 V at the disk to strip $^{dep}Cu$ were applied. Additionally, a 30 second baseline measurement was included in the interval procedure after 3 hours by holding the 0.8 V potential at the disk while setting the ring to 1.2 V. Consequently, no $H_2O_2$ is produced at the disk and only $H_2O_2$ that is already present in the solution is detected at the Pt ring. The results of this measurement are shown in Figure S12 including the schematic overview of the measurement procedure. With the additional ring data, it is clear that $H_2O_2$ is formed at the disk. When the measurement starts, the ring current immediately rises to 50 µA which (with a pre-determined $N_{CE}$ of 18.3% for this measurement) corresponds to a 91% selectivity for $H_2O_2$. However, the ring current decreases to 45 µA and the selectivity drops to 72% over the course of 20 minutes. Every 20 minute cycle, the maximum of the ring current was reached in the first minute of the measurement after which the current decreased. As discussed before, the ring current can also decrease because of $PtO_x$ formation and/or exposure to high amounts of $H_2O_2$. Indeed, the ring current increased to 65 µA after the first interval wherein the potential of the ring was briefly set to 0.55 V demonstrating that the decrease in ring current is mostly caused by these events and not (entirely) by a decrease in selectivity of $H_2O_2$ production. This was found to be the case for every 20 minute measurement. From that, we conclude that $H_2O_2$ is produced throughout the measurement with a high selectivity. Remarkably, 100% $H_2O_2$ production should only lead to a maximum current of 60 µA at the ring. Already in the second 20 minute cycle the maximum ring current is 65 µA. This indicates that $H_2O_2$ from the solution is oxidized as well. The maximum ring current increased every 20 minute cycle (Figure S12B) which is expected if $H_2O_2$ is accumulating in the solution. Hence, a baseline measurement was added to the interval procedure after 3 hours to establish the ring current before $H_2O_2$ is produced at the disk. The baseline current after 3 hours was 50 µA and increased over time to circa 80 µA showing that $H_2O_2$ is accumulating in the solution. Interestingly, the disk current hardly increases and remains stable after 3 hours. Moreover, the increase during each 20 minute cycle is not less pronounced as was found in measurements without the Pt ring electrode (Figures S13 and S14). Most likely, the continuous re-oxidation of a part of the formed $H_2O_2$ and the $H_2O_2$ from the solution will limit the concentration of $H_2O_2$ in the solution. In turn, this would indicate that high $H_2O_2$ concentrations would lead to faster $^{dep}Cu$ formation and more over-reduction of $H_2O_2$ thereby increasing the current rapidly during each 20 minute cycle.

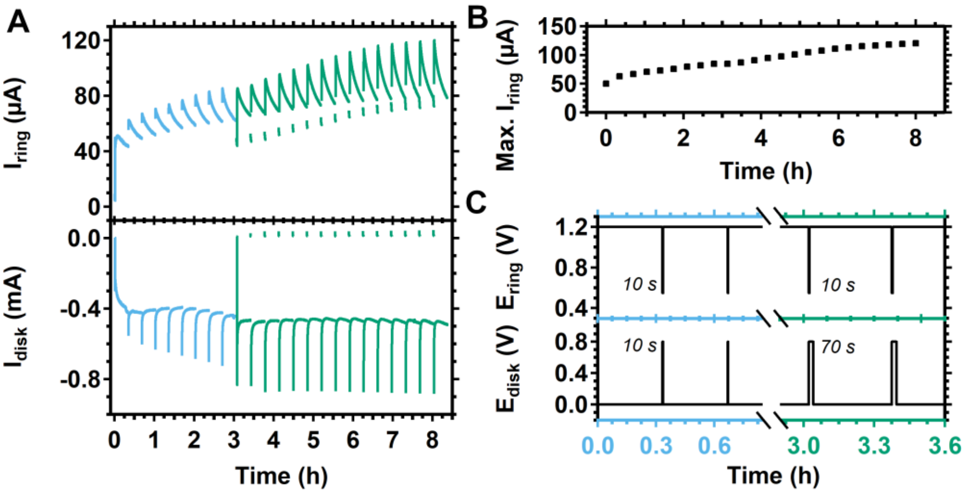


***Figure S12.*** *RRDE chronoamperometry measurement of $O_2$ reduction at 0.0 V by* ***$Cu_2$(btmpa)*** *over the course of 8.5 hours with an interval every 20 minutes (A) with the disk (bottom) and ring current (top). The maximum ring current reached every 20 minutes is depicted in B and the potential versus time plot in C for the blue and green marked areas of A. During the short interval (10 seconds), accumulated $PtO_x$ on the ring could be reduced and $Cu^0$ on the disk could be stripped by a potential of 0.55 V and 0.8 V, respectively. After 3 hours, the disk potential was kept at 0.8 while the ring was set to 1.2 V for 1 minute to establish a baseline ring current.*

## The influence of $^{dep}Cu$ deposition on the Faradaic efficiency for $H_2O_2$

As described, $^{dep}Cu$ formation can become a problem and negatively affect the Faradaic efficiency over longer periods of $O_2$ reduction. In the main text we describe amperometry experiments for two hours. Here we increased the experimental time to 8 hours. The results are plotted in Figure S13. Notably, the Faradaic efficiency decreases over time in both the continuous as well as the interval measurement. The efficiency of the continuous measurement is lower than the interval measurement, apart from the first 30 minutes. In these first 30 minutes, the Faradaic efficiency for $H_2O_2$ is high: 80% for the continuous and 62% for the interval-experiment. The lower Faradaic efficiency for the interval experiment could be explained from the high current noise in these 30 minutes. The measurement suffered from $O_2$ bubbles blocking the surface during continuous purging which resulted in current spikes and a higher overall charge that passed the electrode. After 30 minutes, the set-up of the disk electrode was slightly changed to overcome this problem. As a result, the Faradaic efficiency increased to 69% in the next 30 minutes. This in sharp contrast to the continuous experiment. There, the efficiency dropped to 39%. 1.5 hours after the start, it dropped even further to 31% while the interval measurement still had an efficiency of 63%. Clearly, the 0.8 V interval could strip away $^{dep}Cu$ thereby keeping the Faradaic efficiency high. This is also reflected in the disk current which was lower for the interval experiment than for the continuous experiment. The latter used more charge for the full 4 electron reduction to water. Nonetheless, the Faradaic efficiency dropped significantly for the interval experiment as well after 2 hours. After 6 hours, the calculated efficiency was negative because there had been a decrease in the $H_2O_2$ bulk concentration. This happened for the continuous measurement already after 5 hours. When the calculated selectivity is negative, more $H_2O_2$ is converted to $H_2O$ than that $H_2O_2$ is produced from $O_2$. For the continuous experiment this was expected because of the slow build-up of $^{dep}Cu$, but not for the interval experiment. The 30 second 0.8 V interval was apparently not enough to strip all the formed $^{dep}Cu$. Therefore, the interval time was increased to 4 minutes after 6 hours of amperometry (marked by the grey area in Figure S13). As a result, the Faradaic efficiency went back to a positive value of 6% and the disk current decreased. The bulk $H_2O_2$ concentration went up from 15.6 to 16.7 mg/l which is 0.5 mM of $H_2O_2$. Applying 0.8 V for 4 minutes from the start of the experiment yielded the best result as the Faradaic efficiency remained positive throughout the 8 hour experiments (Figure S14). Overall, these experiments show that $^{dep}Cu$ deposition affects the Faradaic efficiency but this can be counteracted by applying a potential at which $^{dep}Cu$ will strip from time to time.

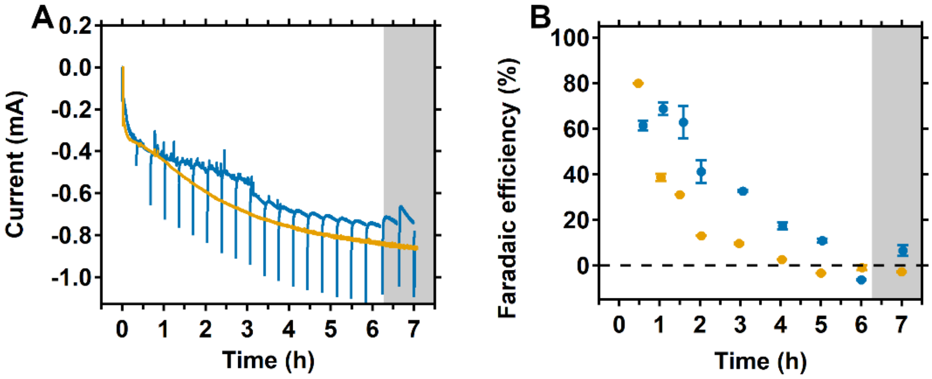


***Figure S13.*** *Rotating disk measurement at 0.0 V of $O_2$ reduction by **$Cu_2$(btmpa)** over the course of 7 hours (A) and the corresponding Faradaic efficiency (B). Each datapoint is the efficiency of the period since the preceding datapoint. A continuous measurement (orange) and an interval measurement (blue) were monitored. A potential of 0.8 V was briefly applied during the intervals. The spikes are an artefact of (re-)applying 0.0 V. In the grey area, the 0.8 V interval time was extended to 4 minutes. The same conditions as Figure 3 were used.*

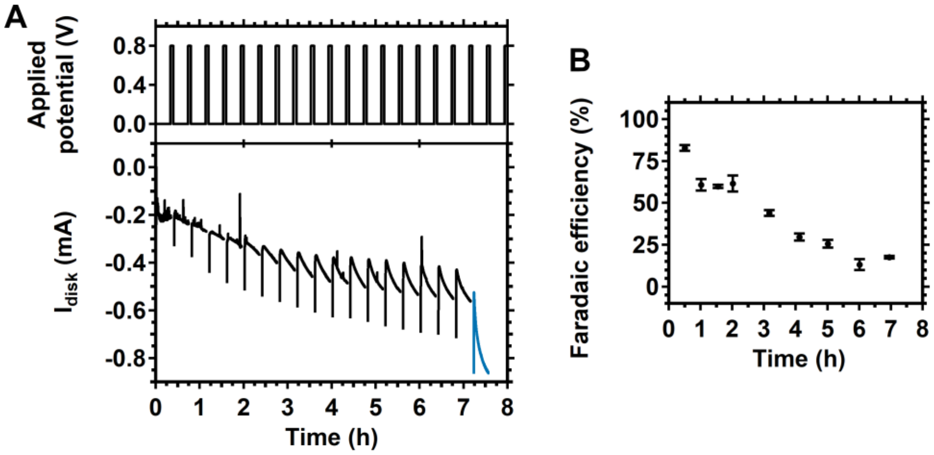


*__Figure S14.__ Rotating disk measurement at 0.0 V of $O_2$ reduction by __$Cu_2$(btmpa)__ (A) and the corresponding Faradaic efficiency (B). The full 8 hours of the measurement of Figure 4.6 is depicted. Before the last 20 minutes (blue line in A), the $H_2O_2$ concentration was manually increased 5 times.*

*__Table S1.__ The concentration of $H_2O_2$ as measured by the enzyme based photometric method for Figure S14. The volume of the solution changed continuously during the measurement as aliquots were taken. Errors are as standard errors.*

| **Hours after start** | **Faradaic efficiency[a] (%)** | **Concentration $H_2O_2$ (mM)** | **Volume of electrolyte (ml)** |
|---|---|---|---|
| 00:30 | 83 ± 2 (3) | 0.040 ± 0.001 | 33.6 |
| 01:01 | 61 ± 3 (3) | 0.077 ± 0.003 | 32.0 |
| 01:33 | 60 ± 1 (3) | 0.124 ± 0.001 | 30.7 |
| 02:01 | 62 ± 5 (3) | 0.166 ± 0.006 | 29.2 |
| 03:10 | 44 ± 2 (3) | 0.273 ± 0.007 | 27.6 |
| 04:07 | 30 ± 2 (4) | 0.340 ± 0.009 | 26.2 |
| 05:01 | 26 ± 3 (4) | 0.41 ± 0.01 | 24.9 |
| 06:01 | 13 ± 3 (5) | 0.45 ± 0.02 | 23.4 |
| 06:57 | 18 ± 1 (3) | 0.503 ±0.005 | 21.9 |

*[a]The Faradaic efficiency corresponds to the period between measurements, not the total Faradaic efficiency since the start of the measurement. Number between brackets is the amount of measurements that was performed.*

Even the long stripping interval of 4 minutes did not completely prevent over-reduction of $H_2O_2$ after 2 hours of amperometry. We believe that this is due to a steady rise in the bulk $H_2O_2$ concentration during the measurement. The rate of $H_2O_2$ over-reduction by **$Cu_2$(btmpa)** will increase with increasing $H_2O_2$ concentration as pointed out earlier (Figure S9). However, it is more likely that that the over-reduction is mostly caused by $^{dep}$Cu as this deposits in a faster rate at higher bulk $H_2O_2$ concentrations. The following observations support this. First of all, the Faradaic efficiency is consistently lower for measurements with shorter or no stripping intervals (Figure S13 and S14) after 2 hours. Also, 2 hours after the start of the measurement, all interval measurements display steeply rising reductive currents during the 20 minutes 0.0 V measurement but all fall back after the stripping interval. These observations point to the accumulation of a species on the electrode ($^{dep}$Cu) that facilitates over-reduction during the 20-minute period which is subsequently stripped after the stripping interval. As mentioned before, **$Cu_2$(btmpa)** itself seems to be more reluctant to desorb from the surface at 0.8 V. Another observation is the enhancement of the steep increase in current when the $H_2O_2$ concentration is manually spiked (Figure S14). Moreover, this steep increase in current is mostly avoided when the formed $H_2O_2$ is intercepted, as was the case in the RRDE experiment (Figure S12). The Pt ring electrode, while sensing $H_2O_2$, is actually continuously converting $H_2O_2$ back to $O_2$ thereby limiting $H_2O_2$ build-up.

To some extent, there is build-up of $H_2O_2$ in the solution, but the reductive disk current remained remarkably constant over the course of 8 hours as opposed to the other measurements and remained close to the theoretical diffusion limited current of –0.49 mA for $O_2$ to $H_2O_2$ reduction. Furthermore, the ring current measurements did indicate that $H_2O_2$ selectivity remained high throughout the measurement. We therefore conclude that the decrease in Faradaic efficiency after 2 hours is caused by higher rates of $^{dep}Cu$ formation induced by a higher $H_2O_2$ concentration.

# The reaction products of $Cu_2$(btmpa) with $H_2O_2$

The faster formation of $^{dep}$Cu after two hours as compared to the start of the measurement might be related to a faster rate of complex degradation. The catalyst-containing electrolyte slowly changes color from blue to green throughout the measurement (Figure S15B). The same spectral changes in the UV-vis spectrum of the electrolyte could be replicated by adding 1.1 mM $H_2O_2$ to a 0.15 mM **$Cu_2$(btmpa)** solution in phosphate buffer (Figure S15A). Monitoring the UV-vis spectrum over the course of a week showed that the low-intensity absorption at 675 nm remained but a second absorption appears at 359 nm. While this peak increased, another peak at 288 nm decreased and an isosbestic point at 298 nm was observed in between. Since the spectrum only changes upon the addition of $H_2O_2$ (Figure S15C), the possibility of $H_2O_2$ coordination to **$Cu_2$(btmpa)** was further explored. The absorption at 359 nm could indicate a $\mu$-$\eta^2$:$\eta^2$-peroxodicopper(II) structure.[15] Typically, complexes and enzymes with such a side-on peroxo dinuclear copper center have a high intensity absorption between 320 and 380 nm as well as a low intensity absorption between 520 and 610 nm. Both absorptions are ascribed to $\pi^* \rightarrow d_{xy}$ peroxo to $Cu^{II}$ charge transfers.[16] The band at 610 nm in that case is most likely is obscured by the 675 nm absorption of unreacted **$Cu_2$(btmpa)**. The possibility of this core was further investigated by Raman spectroscopy (Figure S16). A low energy O–O stretching band is typically observed for this core in the range of 730 – 760 $cm^{-1}$.[15-16] A broad signal at 760 $cm^{-1}$ could be observed, but was also observed in the absence of $H_2O_2$. Since the Raman spectra with and without $H_2O_2$ overlap, no evidence for the presence O–O or Cu–O bonds was found. Therefore, the color and spectral changes are most likely due to ligand degradation by oxidation. This would in turn facilitate $^{dep}$Cu formation, especially at higher $H_2O_2$ concentration.

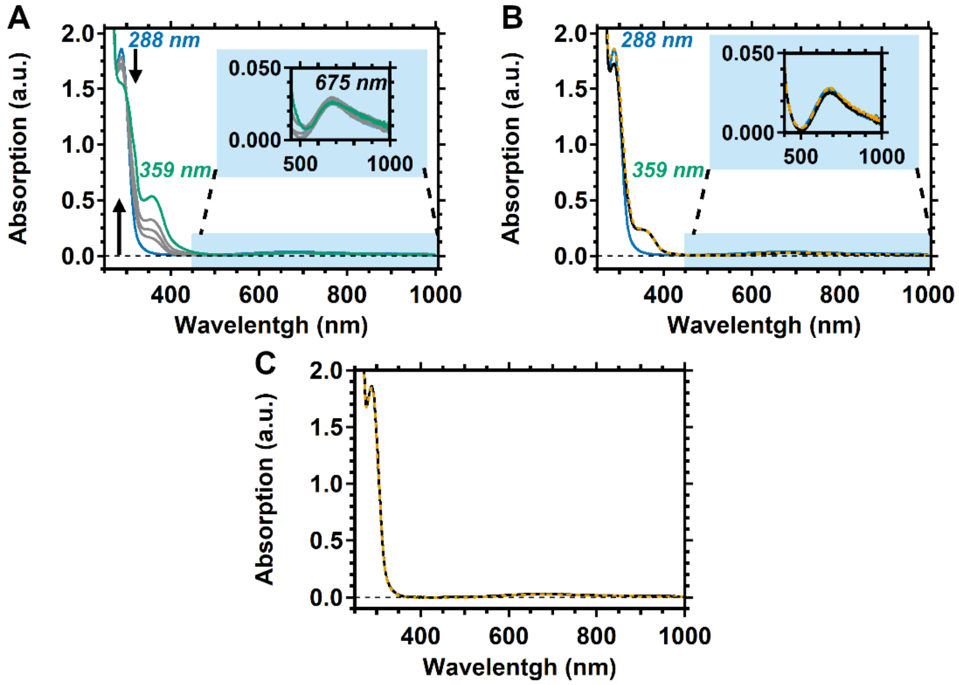


***Figure S15.*** *UV-Vis of 0.15 mM **$Cu_2$(btmpa)** in phosphate buffer. A fresh solution (blue trace) spiked with 1.13 mM $H_2O_2$ was monitored over the course of a week (green trace) (A). B shows a fresh and 8 hours old solution (blue and black trace, respectively) with $H_2O_2$ and an 8 hour old **$Cu_2$(btmpa)** solution after performing $O_2$ reduction at 0.0 V (orange dashed). A fresh (black) and 1 week old solution of **$Cu_2$(btmpa)** (orange dashed) without $H_2O_2$ is shown in C.*

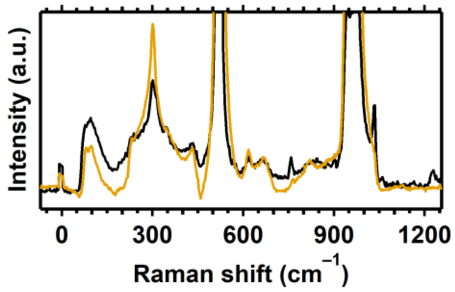


***Figure S16.*** *Raman spectra of a dropcasted solution of 3 mM* ***$Cu_2$(btmpa)*** *(fresh, black) and of a* ***$Cu_2$(btmpa)*** *solution with 22 mM $H_2O_2$ (orange, 9 days old). The solutions were dropcasted on a silicon wafer.*

# XPS Analysis

To assess the presence of Cu deposits at the electrode interface, XPS analysis was carried out on the electrodes after 7 hours of amperometry. Three situations were considered: 1) chronoamperometry with intervals (cf. Figure S13); 2) amperometry without intervals (cf. Figure S13); and 3) amperometry for 30 minutes in presence of complex, followed by 7 hours of amperometry in absence of complex (cf. Figure S11).

After prolonged amperometry at 0.0 V for 7 hours, the GC electrodes were emersed at open circuit potential, rinsed copiously with water, dried under $N_2$ flow and examined with X-ray photoelectron spectroscopy (XPS). Sample shipping and handling were done under ambient air conditions. The Cu $2p_{3/2}$ (A), N 1s (B), and C 1s (C) regions are shown in Figure S17. For reference, the spectrum of the crystalline powder of **$Cu_2$(btmpa)** was recorded as well. In all cases, there is a residue of a copper species left on the electrode after amperometry, as evident from the Cu $2p_{3/2}$ emission lines. Reasonably, the Cu $2p_{3/2}$ signal is less intense after the measurement where **$Cu_2$(btmpa)** was only initially present (spectrum GC 40 min complex, 7 h no complex) in the solution as this probably resulted in less deposit of copper. These electrodes have been continuously exposed to a saturated solution of $O_2$, as well as exposed to air before the XPS measurement was taken. Therefore, only oxidized $Cu^{II}$ BE(Cu $2p_{3/2}$)= 932-933 eV species are observed on the electrode surface and no $Cu^{I}$ or $Cu^{0}$ species. For both the powder reference compound and the Cu containing deposits on the GC electrodes after electrocatalysis, Cu is present in $Cu^{2+}$ state. The N 1s spectrum shows a similar trend for the nitrogen species deposited on the electrode. Again, the major N 1 s species on the electrode surface are slightly more oxidized (major/minor 400.1/398.6 eV) than the nitrogens of the complex (major/minor 399.5/401.2 eV). Also, the composition of the C 1s spectrum has changed but the background of the carbon electrode is hindering any further assignment.

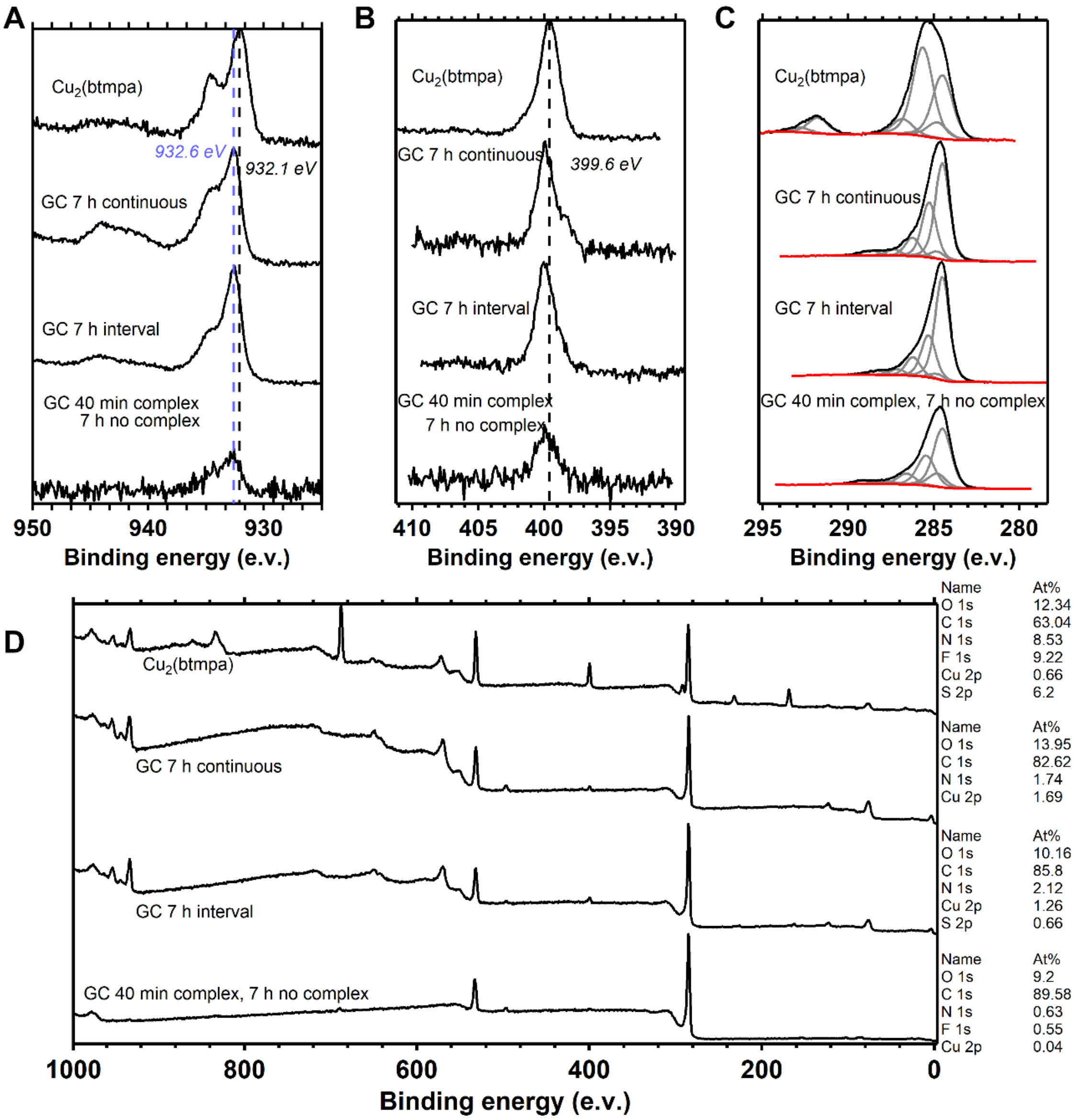


***Figure S17.*** *XP spectra of the Cu 2p (A), N 1s (B), and C 1s (C) regions of the crystalline complex* ***$Cu_2$(btmpa)****, and 3 different GC electrodes. The three different GC electrodes were used for $O_2$ reduction at 0.0 V for several hours in presence of 0.15 mM* ***$Cu_2$(btmpa)****. The continuous and interval experiments were the same as in Figure S13. The last measurement, with only 40 minutes of $O_2$ reduction in presence of the complex, followed by 7 hours of $O_2$ reduction in a blank solution in absence of complex, was the same as the experiment depicted in Figure S11. For the C 1s region (C), the fitted background (in red) and species (grey) used for calibration are shown additionally.*

# References


[1] G. R. Fulmer, A. J. M. Miller, N. H. Sherden, H. E. Gottlieb, A. Nudelman, B. M. Stoltz, J. E. Bercaw, K. I. Goldberg, *Organometallics* **2010**, *29*, 2176–2179.

[2] H. Dürr, K. Zengerle, H.-P. Trierweiler, *Z. Naturforsch. B* **1988**, *43*, 361–368.

[3] F. Forato, A. Belhboub, J. Monot, M. Petit, R. Benoit, V. Sarou-Kanian, F. Fayon, D. Jacquemin, C. Queffelec, B. Bujoli, *Chem. - Eur. J.* **2018**, *24*, 2457–2465.

[4] V. Ganesan, D. Sivanesan, S. Yoon, *Inorg. Chem.* **2017**, *56*, 1366–1374.

[5] a) W. Li, J.-H. Xie, M.-L. Yuan, Q.-L. Zhou, *Green Chem.* **2014**, *16*, 4081–4085; b) G. R. Newkome, D. K. Kohli, F. Fronczek, *J. Chem. Soc., Chem. Commun.* **1980**, *1*, 9–11.

[6] D.-H. Lee, N. N. Murthy, K. D. Karlin, *Inorg. Chem.* **1997**, *36*, 5785–5792.

[7] E. Garribba, G. Micera, *J. Chem. Educ.* **2006**, *83*, 1229.

[8] K. D. Karlin, J. C. Hayes, S. Juen, J. P. Hutchinson, J. Zubieta, *Inorg. Chem.* **1982**, *21*, 4106–4108.

[9] M. Langerman, D. G. H. Hetterscheid, *Angew. Chem. Int. Ed.* **2019**, *58*, 12974–12978.

[10] R. S. Nicholson, *Anal Chem* **1965**, *37*, 1351.

[11] a) N. D. Schley, J. D. Blakemore, N. K. Subbaiyan, C. D. Incarvito, F. D'Souza, R. H. Crabtree, G. W. Brudvig, *Journal of the American Chemical Society* **2011**, *133*, 10473-10481; b) C. J. M. van der Ham, F. Işık, T. W. G. M. Verhoeven, J. W. Niemantsverdriet, D. G. H. Hetterscheid, *Catal. Today* **2017**, *290*, 33–38; c) B. van Dijk, J. P. Hofmann, D. G. H. Hetterscheid, *Phys. Chem. Chem. Phys.* **2018**, *20*, 19625–19634; d) B. van Dijk, G. M. Rodriguez, L. Wu, J. P. Hofmann, A. Macchioni, D. G. H. Hetterscheid, *ACS Catalysis* **2020**, *10*, 4398-4410.

[12] C. Song, J. Zhang, in *PEM Fuel Cell Electrocatalysts and Catalyst Layers: Fundamentals and Applications* (Ed.: J. Zhang), Springer London, London, **2008**, pp. 89–134.

[13] a) P. N. Ross, P. C. Andricacos, *J. Electroanal. Chem. Interfacial Electrochem.* **1983**, *154*, 205–215; b) Y. Zhang, G. S. Wilson, *J. Electroanal. Chem.* **1993**, *345*, 253–271; c) S. B. Hall, E. A. Khudaish, A. L. Hart, *Electrochim. Acta* **1999**, *44*, 4573–4582; d) S. B. Hall, E. A. Khudaish, A. L. Hart, *Electrochim. Acta* **2000**, *45*, 3573–3579; e) S. A. G. Evans, J. M. Elliott, L. M. Andrews, P. N. Bartlett, P. J. Doyle, G. Denuault, *Anal. Chem.* **2002**, *74*, 1322–1326; f) I. Katsounaros, W. B. Schneider, J. C. Meier, U. Benedikt, P. U. Biedermann, A. A. Auer, K. J. J. Mayrhofer, *Phys. Chem. Chem. Phys.* **2012**, *14*, 7384–7391; g) K. Jiang, S. Back, A. J. Akey, C. Xia, Y. Hu, W. Liang, D. Schaak, E. Stavitski, J. K. Nørskov, S. Siahrostami, H. Wang, *Nat. Commun.* **2019**, *10*, 3997.

[14] S. B. Hall, E. A. Khudaish, A. L. Hart, *Electrochim. Acta* **1998**, *43*, 579–588.

[15] L. M. Mirica, X. Ottenwaelder, T. D. P. Stack, *Chem. Rev.* **2004**, *104*, 1013–1046.

[16] a) N. Kitajima, Y. Moro-oka, *Chem. Rev.* **1994**, *94*, 737–757; b) P. K. Ross, E. I. Solomon, *J. Am. Chem. Soc.* **1990**, *112*, 5871–5872; c) P. K. Ross, E. I. Solomon, *J. Am. Chem. Soc.* **1991**, *113*, 3246–3259; d) E. I. Solomon, F. Tuczek, D. E. Root, C. A. Brown, *Chem. Rev.* **1994**, *94*, 827–856.
